# Perceptions of ultra-processed foods, food processing and food healthfulness among a cross-sectional national sample of US adults: do perceptions align with the evidence?

**DOI:** 10.1017/S136898002610233X

**Published:** 2026-03-26

**Authors:** Anna Claire Tucker, Euridice Martínez-Steele, Daphne Abigail Barquera-Guevara, Cindy W. Leung, Lindsey Smith Taillie, Julia A. Wolfson

**Affiliations:** 1 Department of International Health, Johns Hopkins Bloomberg School of Public Health, Baltimore, MD, USA; 2 Center for Epidemiological Studies in Health and Nutrition, University of São Paulo, São Paulo, Brazil; 3 Department of Nutrition, School of Public Health, University of São Paulo, São Paulo, Brazil; 4 Department of Nutrition, Harvard T.H. Chan School of Public Health, Boston, MA, USA; 5 Carolina Population Center, University of North Carolina at Chapel Hill, Chapel Hill, NC, USA; 6 Department of Nutrition, Gillings School of Global Public Health, University of North Carolina at Chapel Hill, Chapel Hill, NC, USA; 7 Department of Health Policy and Management, Johns Hopkins Bloomberg School of Public Health, Baltimore, MD, USA

**Keywords:** NOVA, Food Compass, Consumer awareness, Healthiness perception, Food processing, Ultraprocessed foods, Survey

## Abstract

**Objective:**

There is growing public health interest in ultra-processed foods (UPF), but limited research exploring consumers’ perceptions of these foods in the United States. We aimed to characterise consumers’ beliefs about UPF, the association between perceived food processing and perceived food healthfulness and alignment between consumers’ perceptions and objective measures of food processing and healthfulness.

**Design:**

In a cross-sectional survey, participants answered questions regarding their beliefs about UPF. They rated the healthfulness and processing levels of a random selection of ten out of forty possible foods. We used descriptive statistics to examine participant beliefs about UPF. We used linear regression models to test associations between perceived processing and perceived healthfulness and between objective and perceived measures of food healthfulness and processing.

**Setting:**

We fielded an online survey in the USA in November 2023.

**Participants:**

This study included US adults aged ≥ 18 years (*n* 4455).

**Results:**

Fifty-four percent of participants correctly identified UPF as ‘Food products submitted to a series of industrial processing’ and 52 % correctly identified UPF as, ‘Food products that contain artificial ingredients’. However, one-third of participants believed UPF were genetically modified products. While foods with higher perceived processing tended to have lower perceived healthfulness and individuals perceived UPF as more processed and less healthful than minimally processed foods, healthfulness perceptions better aligned with Food Compass 2.0, a measure that integrates food processing and nutrient-profile.

**Conclusions:**

Educational and policy efforts (e.g. food labeling) are needed to help consumers distinguish UPF and holistically assess the healthfulness of foods and beverages.

Food processing is increasingly considered a key dimension of food healthfulness. Broadly, food processing refers to any deliberate change to an agricultural commodity from origin to point of consumption^(1)^. This can include simple processing such as washing and chopping to more complex processing including canning and pasteurisation^(1)^. Such methods have been used for centuries to improve food safety, preservation, nutritional value and palatability. However, the emergence of industrial food production has resulted in novel food processing methods including extrusion, pre-frying, hydrogenation and the use of cosmetic additives which further optimise foods for abundance, affordability, convenience, shelf life, appearance and palatability^(2,3)^. To describe and differentiate these industrially produced foods, scientists at the University of São Paulo in Brazil introduced the Nova classification system in 2009, which categorises foods according to the extent and purpose of processing^(4)^. This system includes a category for ultra-processed foods (UPF), or industrially produced foods that are a formulation of ingredients, including cosmetic additives and/or substances of little to no culinary use, that are subjected to a series of industrial processes^(5)^.

Over the last decade, a growing body of research has demonstrated associations between intake of UPF and increased risk of cardiometabolic disease. A review by Lane et al found convincing observational evidence that greater intake of UPF is associated with increased risks of incident CVD mortality, type 2 diabetes and some mental disorders^(6)^. Meanwhile, experimental evidence supports that UPF could contribute to obesity through promoting excessive energy intake^(7,8)^. With an increasing global burden of cardiometabolic disease, there is considerable public health interest in UPF and emerging efforts to reduce intake of these foods. Several countries, including Belgium, Brazil, Ecuador, Israel, Maldives, Peru and Uruguay, have already instituted national dietary recommendations to reduce intake of UPF^(9)^.

However, there are several factors that may reduce the effectiveness of these recommendations. First, recommendations can only be effective if consumers can correctly identify UPF. Prior studies among Uruguayan and Brazilian adults found that consumers qualitatively describe UPF similarly to Nova and they can correctly identify many foods as UPF^(10,11)^. Nevertheless, consumers in these countries also identified some non-UPF as UPF (e.g. canned foods, milk, unprocessed meat and even some fruits and vegetables), indicating challenges with UPF identification^(10,11)^.

Another factor that may reduce the effectiveness of recommendations is confusion about the health risks of UPF. Understanding health risks is a key motivator for behaviour change. While many UPF may already be perceived as unhealthy due to their high added sugar, sodium and saturated fat content, other UPF may be perceived as healthy. For example, consumers may perceive whole wheat bread and flavored yogurt as healthy due to food industry marketing and public health guidance to eat more whole grains and dairy. Such discrepancies may create confusion about the health risks of UPF and reduce consumers’ ability to confidently interpret and implement dietary recommendations about UPF and about nutrition more broadly.

While recent studies have made important contributions in characterizing consumers’ perceptions of UPF in Europe and South America^(10–13)^, there is a paucity of research in the US. UPF have been a significant topic of discussion, among US policymakers, public health authorities and in media discourse^(14–16)^, including recent recommendations in the 2025–2030 Dietary Guidelines for Americans to avoid “highly processed” foods^(17)^. Given this, investigating UPF perceptions in the US is vital as consumer perceptions may influence the effectiveness of any policy, public health, or consumer-led movements to reduce UPF intake. Accordingly, this study sought to address the following objectives among a national sample of US adults: (1) describe consumer beliefs about UPF and differences across demographic groups, (2) examine the association between perceptions of food processing and perceptions of food healthfulness and (3) evaluate how consumers’ perceptions relate to objective measures of food processing and healthfulness.

## Methods

We fielded a national online survey using CloudResearch Prime Panels, with adults in the US aged ≥ 18 years in November 2023. To obtain a sample generally reflective of the US population, the survey included gender, race, ethnicity and age distribution quotas based on the 2019–2020 US Census. Survey questions are included in Table A.1.

Of the 9835 individuals who started the survey, we removed individuals who were < 18 years old (*n* 63); did not live in the US (*n* 88); did not provide consent (*n* 1050); were identified as bots, duplicates or had a Qualtrics fraud score ≥ 30 (*n* 555); completed < 75 % of the survey (*n* 965) or failed one or more of the attention checks (*n* 2227).The first attention check included the following instructions: (1) ‘For this item, please mark the response “somewhat agree” to show us you are following directions’ and (2) ‘For this item, please mark the response “strongly disagree” to show us you are following directions’. Responses were given on a Likert scale. For the second attention check, participants were asked, ‘Which of the following is NOT a colour?’ Responses included: red, purple, yellow, penguins, green. We also excluded those who completed the survey in < 10 min (*n* 333), given that the median completion time was 20 min prior to excluding those with completion times < 10 min. After further removing participants who were missing responses related to socio-demographics or UPF perceptions (*n* 99), the final analytic sample was *n* 4455.

### Participant beliefs about ultra-processed foods

Participants were asked, ‘As best you know, ultra-processed foods are’, and they could choose one or more of the following options: (1) ‘Foods composed with more than five ingredients’ (2) ‘Food products submitted to a series of industrial processing’ (3) ‘Genetically modifie products’ (4) ‘Food products that contain artificial ingredients’ and (5) ‘I don’t know what ultra-processed foods are’. Participants were also asked, ‘In your opinion, how healthy or unhealthy are ultra-processed foods?’ Responses were given on a seven-point Likert scale from ‘Not healthy at all’ to ‘Very healthy’. For analysis, these were condensed into the following categories: (1) Not healthy (not at all healthy, unhealthy and slightly unhealthy), (2) Neither healthy or unhealthy and (3) Healthy (slightly healthy, healthy and very healthy).

### Food rating task: participant perceptions of processing and healthfulness

We also designed a task using forty foods and beverages across a range of processing and healthfulness levels. We matched each of these forty foods and beverages to the closest available food code in the 2017–2018 Food and Nutrient Database for Nutrient Studies (FNDDS). Table A.2 lists each food and beverage and the FNDDS food code to which the item was matched. Participants were shown a random sample of ten out of the forty possible foods and beverages. Each item shown to participants included an image and brief description (e.g. homemade sugar cookie), which are included in Table A.1. To avoid biasing responses based on brand preferences, we used generic unbranded items. Ingredients and Nutrition Facts labels were not presented with items.

As participants viewed a random sample of ten of the forty possible items, they were asked, ‘Please rank the healthfulness and degree of processing of this food on a scale from 0–10’. There was a sliding scale from 0–10 for healthfulness, where 0 corresponded to ‘Not at all healthy’ and 10 corresponded to ‘Very healthy’. There was a separate sliding scale from 0–10 for degree of processing, where 0 corresponded to ‘Not at all processed’ and 10 corresponded to ‘Very processed’.

### Food processing: Nova classification

Food processing of foods and beverages from the food ordering task was assessed using Nova. Nova is a classification system that categorises foods according to the extent and purpose of processing. Nova classifies foods into one of four categories: unprocessed and minimally processed foods (Group 1); processed culinary ingredients (Group 2); processed foods (Group 3) and ultra-processed foods (Group 4). Group 1 foods include unprocessed foods such as whole fruits and vegetables, as well as foods that have undergone minimal processing such as grinding, refining, drying and freezing. Group 2 foods include processed culinary ingredients such as sugar, salt and some cooking fats. Group 3 includes manufactured foods that are combinations of Group 1 and Group 2 ingredients. Lastly, UPF are industrial formulations of ingredients including substances not commonly used in culinary preparations and/or cosmetic additives. Foods and beverages in FNDDS have been previously classified according to Nova^(18)^. Briefly, each food code is assigned to a Nova category, or it is represented as a proportion of one or more Nova categories. For potential homemade recipes, the underlying SR codes provided by FNDDS are used to estimate Nova proportions. Nova categories for all forty foods and beverages used in this survey are presented in Table A.3.

For the purpose of this study, we classified items shown to participants into one of three categories: minimally processed and home-prepared foods; processed foods and UPF. While Nova categories differ from the way we assessed perceived processing on a continuous scale, we felt it was important to be able to capture gradations in participants’ perceptions of processing. While this may have introduced a mismatch, it also allowed us to explore the relationship between perceptions and Nova regardless of consumers’ familiarity with Nova.

### Food healthfulness: Food Compass 2.0

We additionally scored foods and beverages from the food ordering task using Food Compass 2.0. Food Compass 2.0 is a nutrient profiling system used to rank the healthfulness of foods and beverages from 0 to 100 based on several characteristics including nutrient-based factors (nutrient ratios, vitamins, minerals, lipids, fibre, protein and phytochemicals) and food processing factors (ingredients, additives and Nova). As foods and beverages in FNDDS have been previously assigned Food Compass 2.0 scores, we used the scores available in the Supplementary Tables of Barrett *et al.* ^(19)^ Using the cut points specified by Barrett et al, we further classified foods and beverages into one of three categories: foods to encourage (Food Compass score: ≥ 70); foods to moderate (Food Compass score: 31–69) and foods to minimise (Food Compass score: ≤ 30). Food Compass 2.0 scores for all forty foods and beverages are also presented in Table A.3.

### Socio-demographic variables

The survey asked participants about socio-demographic characteristics including gender (male, female and non-binary, other); age in years (< 18, 18–29, 30–39, 40–49, 50–59, 60–69, 70–79, 80–89 and 90+); race (Asian, Black or African American, Native American, Middle Eastern or North African, Pacific Islander, White/Caucasian and Other – please describe); Hispanic, Latino, or Spanish ethnicity (Yes/No); income (< $25 000, $25 000–$49 999, $50 000–$74 999, $75 000–$99 999, $100 000–$149 999, $150 000 or more, prefer not to say); education (some high school, high school diploma or GED, some college, college degree and graduate degree); political affiliation (Republican, Democrat or Independent) and household size (1, 2, 3, 4, 5, 6, 7 or 8 or more). Due to small sample sizes in some categories, the following variables were modified: gender (non-binary and other were combined); age (70–79, 80–89 and 90+ were combined); race (Middle Eastern or North African, Pacific Islander and Other were combined); income (< $25 000, $25 000–$74 999, ≥ $75 000, or prefer not to say); education (high school or less, some college, college degree or higher) and household size (1 person, 2–3 people or 4 or more people).

### Statistical analysis

We used descriptive statistics and *χ* ^2^ tests to examine participant beliefs about UPF stratified by participant demographic characteristics (age, gender, race, ethnicity, income, education, political affiliation and household size). Additionally, for each of the forty foods and beverages, we used descriptive statistics to estimate mean perceived processing scores and mean perceived healthfulness scores, only using scores provided by participants who viewed a given item.

To test the association between perceived processing (quartiles) and perceived healthfulness (continuous from 0 to 10), we used linear regression models with individual fixed effects and standard errors clustered by food item. This analytic approach addresses non-independence of observations, accounting for repeated ratings by the same individual and clustering of ratings at the food level^(20)^. Additionally, because fixed effects restrict to within-person comparisons, this approach accounts for observed and unobserved characteristics, such as demographic characteristics or an individual’s tendency to systematically rate all foods as more or less healthy^(21)^. Thus, further adjustment for covariates is unnecessary as these are inherently dropped from a model with individual fixed effects. To improve interpretability, perceived processing was modelled as quartiles, which were generated by ranking perceived processing scores. The same analytic approach was used to additionally examine the following associations: Nova category (minimally processed and home-prepared foods, processed foods and UPF) and perceived processing score (continuous); Nova category and perceived healthfulness score (continuous); Food Compass score category (foods to encourage, foods to moderate and foods to minimise) and perceived processing score (continuous) and Food Compass score category and perceived healthfulness score (continuous). Predicted means were obtained using the post-estimation margins command following each model.

All analyses were conducted using Stata, version 18. Tests were two-sided, and statistical significance was set at *P* < 0·05.

## Results

Table 1 presents beliefs about ultra-processed food healthfulness overall and stratified by demographic characteristics. Overall, the majority (73·8 %) of participants believed that UPF were not healthy, but responses varied across demographic groups. Specifically, men, adults < 40 years, Black or African American, Native American, those with household income < $25 000, high school education or less and households with ≥ 4 people were less likely to think UPF were unhealthy.

**Table 1.** Beliefs about ultra-processed food healthfulness overall and stratified by demographic characteristics (*n* 4455)

			In your opinion, how healthy or unhealthy are ultra-processed foods?^*^						
	*n*	%	Not healthy		Neither healthy nor unhealthy		Healthy		*P* value
			*n*	%	*n*	%	*n*	%	
Overall	4455		3287	73·8 %	861	19·3 %	303	6·8 %	
Gender									
Male	2029	45·5 %	1408	69·4	424	20·9	197	9·7	< 0·0001
Female	2391	53·7 %	1848	77·4	433	18·1	106	4·4	
Non-binary or other	35	0·8 %	31	88·6	4	11·4	0	0·0	
Age									
18–29	637	14·3 %	445	69·9	126	19·8	66	10·4	< 0·0001
30–39	647	14·5 %	451	69·8	121	18·7	74	11·5	
40–49	739	16·6 %	542	73·3	128	17·3	69	9·3	
50–59	949	21·3 %	751	79·2	157	16·6	40	4·2	
60–69	675	15·2 %	508	75·4	139	20·6	27	4·0	
70 and older	808	18·1 %	590	73·1	190	23·5	27	3·4	
Race and Ethnicity									
White	2890	64·9 %	2161	74·8	553	19·2	174	6·0	< 0·0001
Asian	158	3·5 %	121	76·6	27	17·1	10	6·3	
Black or African American	504	11·3 %	320	63·5	132	26·2	52	10·3	
Native American	56	1·3 %	36	64·3	18	32·1	2	3·6	
Hispanic	671	15·1 %	512	76·5	98	14·7	59	8·8	
Other	176	4·0 %	137	77·8	33	18·8	6	3·4	
Income									
< $25 000	944	21·2 %	637	67·5	251	26·6	56	5·9	< 0·0001
$25 000–< $75 000	1979	44·4 %	1488	75·3	382	19·3	106	5·4	
≥ $75 000	1372	30·8 %	1043	76·1	193	14·1	135	9·9	
Prefer not to say	160	3·6 %	119	74·4	35	21·9	6	3·8	
Education									
High school or less	1150	25·8 %	761	66·2	307	26·7	81	7·1	< 0·0001
Some college	1367	30·7 %	1043	76·4	261	19·1	62	4·5	
College degree or higher	1938	43·5 %	1483	76·6	293	15·1	160	8·3	
Political affiliation									
Republican	1360	30·5 %	1018	71·8	266	17·0	74	11·2	< 0·0001
Democrat	1581	35·5 %	1134	75·0	269	21·6	177	3·4	
Independent	1514	34·0 %	1135	73·9	326	19·3	52	6·8	
Household size									
1 person	1035	23·2 %	786	75·9	196	18·9	53	5·1	< 0·0001
2–3 people	2407	54·0 %	1801	74·9	462	19·2	142	5·9	
4 or more	1013	22·7 %	700	69·2	203	20·1	108	10·7	

*P*-value from chi-squared test of association between two categorical variables.

* Participants were asked, ‘In your opinion, how healthy or unhealthy are ultra-processed foods?’ Answers ranged from ‘Not at all healthy’ to ‘Very healthy’ on a seven-point Likert scale.

Table 2 includes participant definitions of UPF stratified by age and education, with results across all demographic characteristics presented in Table A.4. Overall, more than half of participants correctly defined UPF as, ‘Food products submitted to a series of industrial processing’ (54·4 %). Likewise, 52·0 % believed UPF are, ‘Food products that contain artificial ingredients’, and 26·4 % believed UPF are ‘Foods composed with more than 5 ingredients’. However, nearly one-third of participants incorrectly identified UPF as, ‘GM products’, while nearly one-quarter (23·6 %) stated they did not know what UPF are. Adults > 40 years and those with a high school education or less were more likely to state they do not know what UPF are. Meanwhile, adults < 40 years were more likely to identify UPF as GM products and were more likely to believe UPF are food products containing artificial ingredients. Those with a high school education or less were less likely to identify UPF as foods submitted to a series of industrial processes or foods containing artificial ingredients.

**Table 2.** Definition of ultra-processed foods among national sample of US adults stratified by age and education (*n* 4455)

	Ultra-processed foods are^*^														
	Foods composed with more than 5 ingredients			Food products submitted to a series of industrial processing			Genetically modified products			Food products that contain artificial ingredients			I don’t know what ultra- processed foods are		
	*n*	%	*P* value	*n*	%	*P* value	*n*	%	*P* value	*n*	%	*P* value	*n*	%	*P* value
Overall	1175	26·4 %		2423	54·4 %		1459	32·7 %		2315	52·0 %		1053	23·6 %	
Age															
18–29	177	27·8	< 0·001	347	54·5	0·027	290	45·5	< 0·001	368	57·8	0·001	131	20·6	< 0·001
30–39	209	32·3		349	53·9		267	41·3		360	55·6		120	18·6	
40–49	216	29·2		378	51·2		267	36·1		397	53·7		165	22·3	
50–59	239	25·2		499	52·6		255	26·9		467	49·2		243	25·6	
60–69	162	24·0		370	54·8		166	24·6		330	48·9		183	27·1	
70 and older	172	21·3		480	59·4		214	26·5		393	48·6		211	26·1	
Education															
High school or less	280	24·4	0·174	488	42·4	< 0·001	366	31·8	0·241	499	43·4	< 0·001	389	33·8	< 0·001
Some college	376	27·5		779	57·0		472	34·5		728	53·3		301	22·0	
College degree or higher	519	26·8		1156	59·7		621	32·0		1088	56·1		363	18·7	

*P*-value from chi-squared test of association between two categorical variables.

* Participants were asked, ‘As best you know, ultra-processed foods are’, and they could choose one or more of the following options: (1) ‘Foods composed with more than five ingredients’ (2) ‘Food products submitted to a series of industrial processing’ (3) ‘Genetically Mmodifie products’ (4) ‘Food products that contain artificial ingredients’ and (5) ‘I don’t know what ultra-processed foods are’.

**Figure 1.**
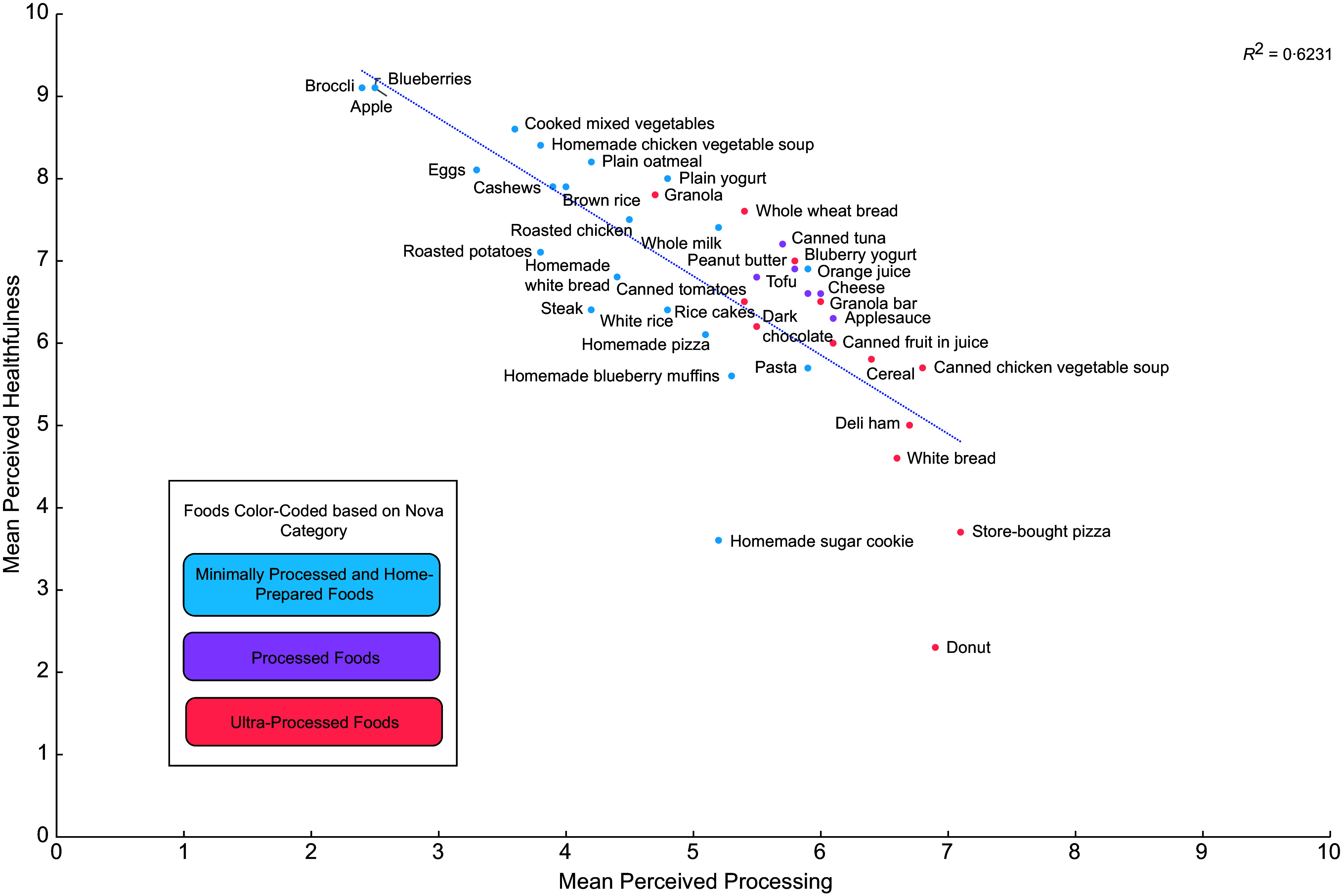
Scatterplot of mean perceived healthfulness and mean perceived processing of forty foods identified by Nova category and scored by a national sample of US adults (*n* 4455)^a^. ^a^For a given food, participants were shown a picture of the food and were asked to rate the healthfulness and degree of processing of each food. Processing and healthfulness were ranked on a scale from 0–10, where 0 corresponded to ‘not at all processed’ or ‘not at all healthy’ and 10 corresponded to ‘very processed’ or ‘very healthy’.

**Figure 2.**
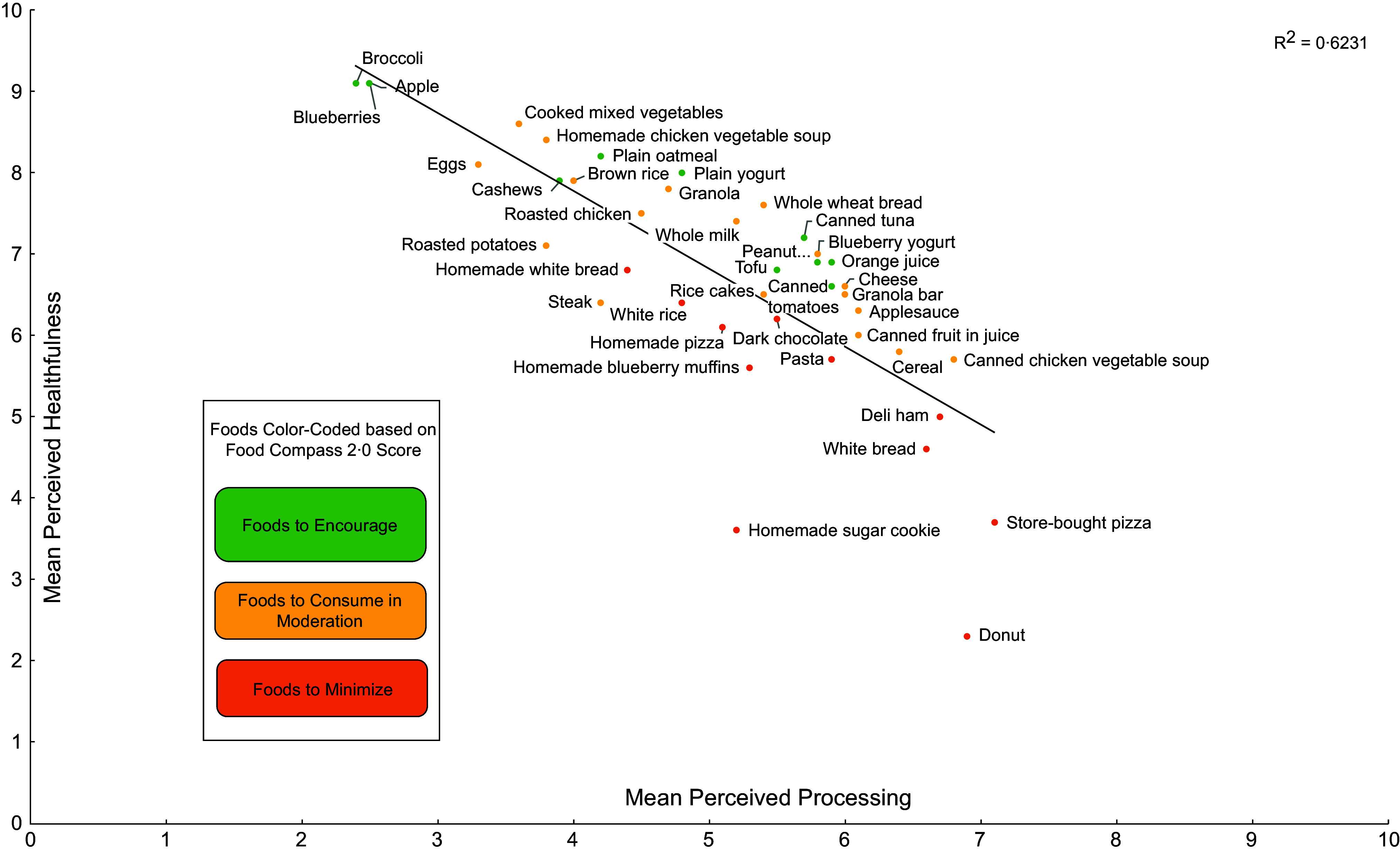
Scatterplot of mean perceived healthfulness and mean perceived processing of forty foods identified by Food Compass 2.0 and scored by a national sample of US adults (*n* 4455)^a^. ^a^For a given food, participants were shown a picture of the food and were asked to rate the healthfulness and degree of processing of each food. Processing and healthfulness were ranked on a scale from 0 to 10, where 0 corresponded to ‘not at all processed’ or ‘not at all healthy’ and 10 corresponded to ‘very processed’ or ‘very healthy’.

Mean perceived processing and mean perceived healthfulness scores for all forty foods shown to participants are listed in Table A.3, with these scores plotted in Figures 1 and 2. As shown in both figures, when examining correlation between these mean scores for each food, there was a strong negative correlation between mean perceived processing and mean perceived healthfulness (*r* = –0·79). Figure 1 identifies each item based on Nova category. With the exception of granola, which had a mean perceived processing score of 4·7 (sd = 2·8), mean perceived processing scores were higher for all UPF compared with nearly all minimally processed foods. Additionally, all but two UPF (i.e. granola and whole wheat bread) had lower mean perceived healthfulness scores than the majority of minimally processed foods.

Figure 2 identifies each item based on Food Compass 2.0. With the exception of homemade white bread, all Foods to Minimise were perceived as less healthful than Foods to Encourage. Perceived processing was less aligned with Food Compass scores. Five of the Foods to Encourage (i.e. tofu, canned tuna, blueberry yogurt, orange juice and canned tomatoes) had higher perceived processing scores than more than half of the Foods to Minimise (i.e. homemade white bread, white rice, homemade pizza, dark chocolate and homemade sugar cookie).

Within-person associations between perceived processing and perceived healthfulness are presented in Table 3, with mean predicted healthfulness ratings reported across quartiles of perceived processing. Among foods in the lowest quartile of perceived processing, mean perceived healthfulness was 7·95 (95 % CI: 7·50, 8·41). Perceived healthfulness decreased across increasing quartiles of perceived processing. Foods in the highest quartile of perceived processing had the lowest mean perceived healthfulness (5·47, 95 % CI: 4·84, 6·10).

**Table 3.** Predicted mean perceived healthiness score across quartiles of perceived processing (*n* 4447)^*^

Perceived processing quartile	Predicted mean perceived healthfulness^†^	95 % CI
Quartile 1^‡^	7·95	7·50, 8·41
Quartile 2	6·74	6·46, 7·02
Quartile 3	6·35	6·08, 6·63
Quartile 4	5·47	4·84, 6·10

* Individuals who only scored one food were dropped from analysis (*n* 8).

† Predicted margins based on post-estimation margins command used after linear regression model with fixed effects for individuals and clustered standard errors by food item.

‡ Minimum and maximum values of perceived processing scores for each quartile: Quartile 1 = 0–3; Quartile 2 = 4–5; Quartile 3 = 6–7 and Quartile 4 = 8–10.

Table 4 presents results from models examining the within-person associations between Nova and Food Compass 2.0 and perceived processing and healthfulness. Mean perceived processing was higher among UPF (6·09, 95 % CI: 5·72, 6·45) compared with minimally processed foods (4·25, 95 % CI: 3·84, 4·66). Likewise, UPF had lower mean perceived healthfulness (5·80, 95 % CI: 5·02, 6·59), compared with minimally processed foods (7·33, 95 % CI: 6·78, 7·88). The difference in mean perceived healthfulness was larger across Food Compass 2.0 categories. Foods to Encourage had a mean perceived healthfulness of 7·78 (95 % CI: 7·21, 8·36) and mean perceived processing of 4·47 (95 % CI: 3·64, 5·30), while Foods to Minimise had mean perceived healthfulness of 5·12 (95 % CI: 4·34, 5·91) and mean perceived processing of 5·76 (95 % CI: 5·22, 6·30).

**Table 4.** Predicted mean perceived processing score and mean perceived healthfulness score across Nova and Food Compass 2.0 categories (*n* 4447)^*^

	Predicted mean perceived processing score^†^	95 % CI	Predicted mean perceived healthfulness score^†^	95 % CI
Nova category				
Minimally processed and home-prepared foods	4·25	3·84, 4·66	7·33	6·78, 7·88
Processed foods	5·82	5·67, 5·97	6·72	6·49, 6·94
Ultra-processed foods	6·09	5·72, 6·45	5·80	5·02, 6·59
Food Compass 2.0^‡^				
Foods to encourage	4·47	3·64, 5·30	7·78	7·21, 8·36
Foods to moderate	5·04	4·54, 5·53	7·10	6·71, 7·48
Foods to minimise	5·76	5·22, 6·30	5·12	4·34, 5·91

* Individuals who only scored one food were dropped from analysis (*n* 8).

† Predicted margins based on post-estimation margins command used after linear regression model with fixed effects for individuals and clustered standard errors by food item.

‡ Food Compass 2.0 categories: Foods to Encourage (FCS 2.0 scores ≥ 70); Foods to Moderate (FCS 2.0 scores > 30 and < 70) and Foods to Minimize (FCS 2.0 scores ≤ 30).

## Discussion

In this national survey of a large sample of US adults, we evaluated beliefs about UPF, examined the association between perceived processing and perceived healthfulness and explored how consumer perceptions relate to objective measures of processing and healthfulness. Most participants correctly identified some characteristics of UPF. However, incorrect beliefs about UPF were common with one-third of participants believing UPF were genetically modified products. The majority of participants believed UPF are unhealthy. Accordingly, we found that greater perceived processing was associated with lower perceived healthfulness and individuals perceived UPF as more processed and less healthful than minimally processed foods, which is consistent with Nova. However, participants’ perceptions of healthfulness may better align with Food Compass 2.0. Findings highlight the need to dispel misconceptions about UPF and ensure consumers are equipped to confidently differentiate between UPF and non-UPF.

More than half of US consumers in our sample believed UPF are ‘Food products submitted to a series of industrial processing,’ which is comparable with estimates from a multinational study of Brazilian, Dutch and Italian consumers^(12)^. However, <40 % of consumers in the multinational study believed UPF are ‘Food products that contain artificial ingredients’, and less than 20 % believed UPF are ‘genetically modified products’^(12)^. In contrast, we found that over half of US consumers believe UPF have artificial ingredients and nearly one-third believed UPF are genetically modified products. Additionally, nearly 24 % of US adults did not know what UPF are compared to <10 % in the multinational study^(12)^. Our results are more comparable with findings from the United Kingdom, where 22 % of adults reported not knowing what UPF are^(22)^. While consumers in Brazil may have greater awareness of UPF due to concerted efforts to reduce UPF consumption by the Brazilian Dietary Guidelines^(23)^, it is notable that a greater proportion of US adults both correctly identified UPF as foods with artificial ingredients and incorrectly identified UPF as genetically modified products. These findings indicate the need for more education among US consumers to increase familiarity with the term and dispel the belief that UPF are genetically modified foods.

Additionally, we found that consumers’ perceptions of food processing are similar to Nova, as mean perceived processing was higher for UPF compared with minimally processed foods. All but one UPF (i.e. granola) had higher perceived processing than nearly all minimally processed foods (i.e. excluding orange juice and pasta). However, Nova Group 3 processed foods (e.g. cheese, peanut butter and applesauce) had similar perceived processing scores to UPF with higher perceived healthfulness (i.e. canned fruit in juice, dark chocolate and rice cakes). It is important to note that these foods (e.g. peanut butter, applesauce and canned fruit in juice) could fall into Nova Group 3 or Nova Group 4, depending on the brand and formulation. Consumers may not be aware of the distinction between food processing that qualifies some foods as Group 3 processed foods, compared with other food processing that qualifies foods as Group 4 UPF. These findings may have implications for how US consumers would implement advice to avoid UPF. For example, if consumers sought to avoid UPF, they might mistakenly avoid non-UPF with higher perceived processing (e.g. tofu, peanut butter, canned tomatoes). Alternatively, they might only avoid foods with the highest perceived processing (i.e. white bread, deli ham, canned chicken vegetable soup, store-bought pizza and donut), while not realising that foods with lower perceived processing (e.g. granola bar, rice cake, whole wheat bread and blueberry yogurt) are also UPF.

In this study of US adults, and in prior studies across South America and Europe, the majority of consumers consider UPF to be unhealthy^(10–12,24,25)^. Accordingly, we found that foods with higher mean perceived processing scores tended to have lower mean perceived healthfulness scores. This reinforces findings from Hassig et al and Bolhius et al, where both studies observed strong food-level correlations between perceived processing and perceived healthfulness^(12,13)^. To see if average food-level correlations held up at the individual level, we examined within-person associations between perceived processing and perceived healthfulness, and found that, on average, individuals who rated foods as more highly processed also rated these foods as less healthful.

However, it is important to note that only 11·8 % of within-person variability in perceived healthfulness across different foods was explained by perceived processing in the model, suggesting that there are additional factors shaping perceived healthfulness, as reported by prior qualitative studies^(25,26)^. While this finding is unsurprising, it is an important consideration in light of an experimental study among Brazilian adults that found that the addition of an UPF label had no effect on purchase intentions^(27)^. Specifically, food processing may have a relatively small impact of perceived healthfulness relative to other factors (e.g. nutrient content, branding and marketing to signal healthfulness) that could have synergistic or competing effects on perceived healthfulness.

Additionally, the myriad factors shaping healthfulness perceptions may explain why there was greater differentiation in mean perceived healthfulness scores across Food Compass 2.0 categories compared with Nova. Nova represents one dimension of healthfulness – food processing – and on its own, this is not sufficient to help consumers build a healthy dietary pattern^(28)^. For example, there are minimally processed foods inherently high in saturated fat that should be limited in the diet (e.g. fatty cuts of red meat), and minimally processed foods may be combined with excessive added sugar, Na and saturated fat in home preparations. Food Compass 2.0, in contrast, is based on several dimensions of healthfulness including food processing, nutrient ratios, carbohydrate quality, key ingredients and presence of phytonutrients and certain additives. Thus, as a more holistic measure, Food Compass 2.0 may better align with consumers’ intuitive understanding of food healthfulness.

Given poor dietary quality among US adults^(29)^ and the high burden of diet-related diseases^(30,31)^, improved strategies are needed to help consumers identify foods that form the foundation of a healthy diet. However, telling consumers to avoid UPF may not have the intended impact on consumers’ food choices, given that one-third of US consumers believe that UPF are genetically modified products and one-quarter do not know what UPF are. Furthermore, some food processing (e.g. pasteurisation, freezing and canning) is important for food safety and does not inherently mean a food is ultra-processed^(32)^, which may be confusing to consumers, as evidenced by Nova Group 3 processed foods scoring similarly on perceived processing as some UPF. While educational efforts or food labeling based on Nova could mitigate these misunderstandings, it may be more difficult to overcome consumers’ perceptions of food healthfulness – including the perceptions that some UPF have healthfulness comparable to minimally processed and processed foods.

Given this, further research is needed to explore how Nova can be communicated to support healthier food choices without unintended consequences (e.g. mistakenly avoiding non-UPF with higher perceived processing), and whether a more holistic measure of food healthfulness that preemptively integrates both food processing and nutrient-based characteristics (i.e. Food Compass 2.0) is easier for consumers to use and understand. Such efforts will be especially critical given the stream of inconsistent media messaging that could further confuse consumers, erode trust in nutrition research and reduce the efficacy of future public health educational campaigns on UPF^(33,34)^.

Strengths of this study include the use of a large, national sample. However, there are also several limitations. First, findings may not be generalisable beyond the 40 foods included in the survey. Additionally, we asked participants to rate food processing on a scale from 0 to 10, rather than asking participants to classify foods into a Nova category. While it seems unlikely, it is possible that participants’ perceptions of processing were poorly aligned with Nova, but participants could have still accurately classified UPF. Additionally, the Nova group assigned to certain food items may not apply uniformly across all products on the market, which could have influenced participants’ responses depending on the specific brands they consume. For example, peanut butter was assumed to be Group 3 because this is how it is classified in FNDDS; although some peanut butters could fall into Group 4 – or even Group 1 – depending on formulation. The use of linear regression models with individual fixed effects limits generalisability of these findings, as between-person variability is eliminated in the analysis. However, it is important to note that within-person associations between perceived processing and healthfulness were consistent with food-level patterns and findings from prior studies. Additionally, the select-all-that-apply question about UPF may not have allowed us to capture the breadth of views that exist about UPF or extent of confusion that might exist among consumers. Future qualitative research could serve to further explore some of the patterns observed in this study.

### Conclusions

While the majority of consumers correctly identified some characteristics of UPF, many participants across demographic groups were not prepared to identify UPF. Additionally, findings indicate food processing may just be one of many factors contributing to perceptions of food healthfulness, and Food Compass 2.0 may align better with consumers’ perceptions of healthfulness compared to Nova. For these reasons, educational and policy efforts are needed to help consumers identify UPF and holistically assess the healthfulness of foods and beverages.

## Supporting information

10.1017/S136898002610233X.sm001 Tucker et al. supplementary material Tucker et al. supplementary material

## Supplementary material

For supplementary material accompanying this paper visit https://doi.org/10.1017/S136898002610233X
