# Supplementary material for: Perceptions of ultra-processed foods, food processing and food healthfulness among a cross-sectional national sample of US adults: do perceptions align with the evidence?
Source: Public Health Nutr. 2026 Mar 26;29(1):e89. doi: 10.1017/S136898002610233X (PMC13112312; doi:10.1017/S136898002610233X)
Supplement: Tucker et al. supplementary material [file S136898002610233Xsup001.docx]

**Table A.1. Palate Pulse Survey Questions**

How old are you?

- Less than 18 years old (9)
- 18-29
- 30-39
- 40-49
- 50-59
- 60-69
- 70-79
- 80-89
- 90+

What is your race? Please select all that apply.

- Asian
- Black or African American
- Native American
- Middle Eastern or North African
- Pacific Islander
- White/ Caucasian
- Other (please describe)

Do you identify as Hispanic, Latino, or of Spanish origin?

- No
- Yes

How many total people live in your household (including yourself, any children, and elderly persons)?

- 1
- 2
- 3
- 4
- 5
- 6
- 7
- 8 or more

How do you identify?

- Male
- Female
- Non-binary
- Other __________________________________________________

In politics, as of today, do you consider yourself a Republican, a Democrat, or an Independent?

- Republican
- Democrat
- Independent

What was your total household income before taxes during the past 12 months?

- Less than $25,000
- $25,000-$49,999
- $50,000-$74,999
- $75,000-$99,999
- $100,000-$149,999
- $150,000 or more
- Prefer not to say

What is the highest level of education you have completed?

- Some high school
- High School diploma or GED
- Some college
- College degree
- Graduate degree

As best you know, ultra-processed foods are: (please check all that apply)

- Foods composed with more than 5 ingredients
- Food products submitted to a series of industrial processing
- Genetically modified products
- Food products that contain artificial ingredients
- I don't know what ultra-processed foods are

In your opinion, how healthy or unhealthy are ultra-processed foods?

- Not healthy at all
- Unhealthy
- Slightly unhealthy
- Neither healthy or unhealthy
- Slightly healthy
- Healthy
- Very healthy

How much do you agree or disagree with the following statement?
**Ultra-processed foods contribute to weight gain.**

- Strongly disagree
- Somewhat disagree
- Neither agree nor disagree
- Somewhat agree
- Strongly agree

Next, you will see pictures of a few different foods.

For each food, please use the sliding scales to rate the healthfulness and degree of processing of each food.

When ranking healthfulness and degree of processing, please use the following criteria:

 **Healthfulness: 0 = not at all healthy. 10 = very healthy.
 Processing: 0 = not at all processed. 10 = very processed.**

**This is deli ham.**


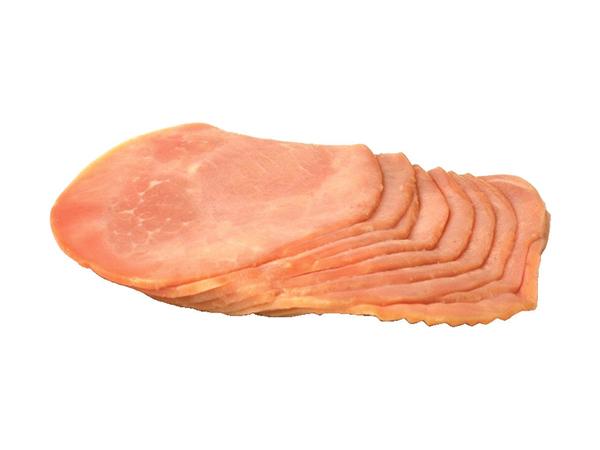


Please rank the healthfulness and degree of processing of this food on a scale from 0-10.
**Healthfulness:** 0 = not at all healthy. 10 = very healthy.
**Processing:** 0 = not at all processed. 10 = very processed.

|  | 0 | 1 | 2 | 3 | 4 | 5 | 6 | 7 | 8 | 9 | 10 |
| --- | --- | --- | --- | --- | --- | --- | --- | --- | --- | --- | --- |

| Healthfulness () | 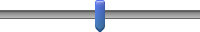 |
| --- | --- |
| Degree of Processing () | 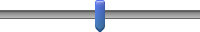 |

**This is white bread.**

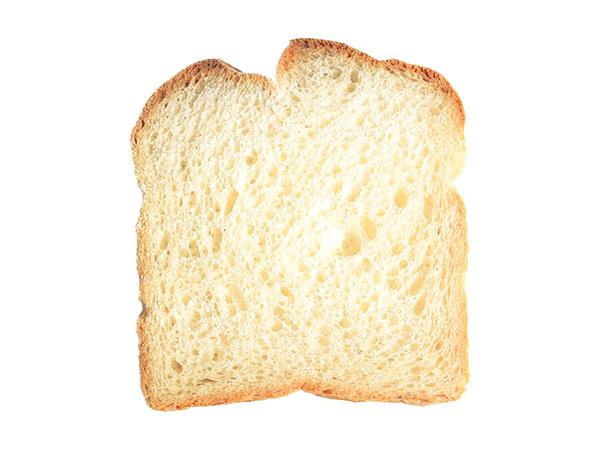

Please rank the healthfulness and degree of processing of this food on a scale from 0-10.
**Healthfulness:** 0 = not at all healthy. 10 = very healthy.
**Processing:** 0 = not at all processed. 10 = very processed.

|  | 0 | 1 | 2 | 3 | 4 | 5 | 6 | 7 | 8 | 9 | 10 |
| --- | --- | --- | --- | --- | --- | --- | --- | --- | --- | --- | --- |

| Healthfulness () | 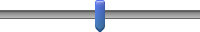 |
| --- | --- |
| Degree of Processing () | 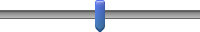 |

**This is a donut.**

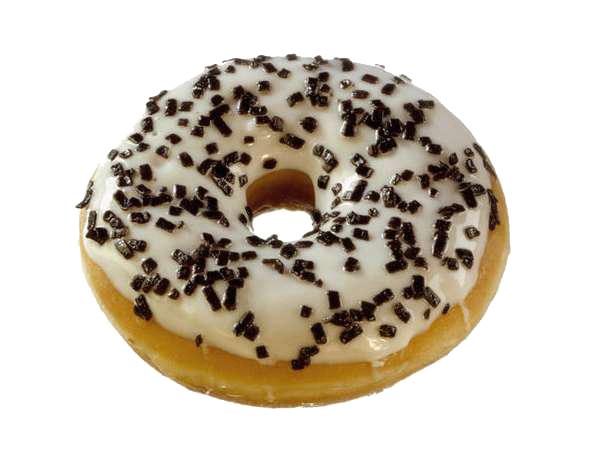


Please rank the healthfulness and degree of processing of this food on a scale from 0-10.
**Healthfulness:** 0 = not at all healthy. 10 = very healthy.
**Processing:** 0 = not at all processed. 10 = very processed.

|  | 0 | 1 | 2 | 3 | 4 | 5 | 6 | 7 | 8 | 9 | 10 |
| --- | --- | --- | --- | --- | --- | --- | --- | --- | --- | --- | --- |

| Healthfulness () | 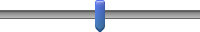 |
| --- | --- |
| Degree of Processing () | 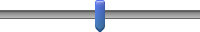 |

**This is store-bought pizza.**

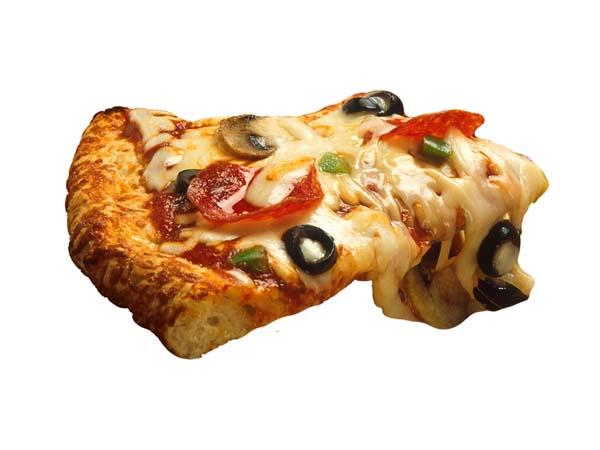


Please rank the healthfulness and degree of processing of this food on a scale from 0-10.
**Healthfulness:** 0 = not at all healthy. 10 = very healthy.
**Processing:** 0 = not at all processed. 10 = very processed.

|  | 0 | 1 | 2 | 3 | 4 | 5 | 6 | 7 | 8 | 9 | 10 |
| --- | --- | --- | --- | --- | --- | --- | --- | --- | --- | --- | --- |

| Healthfulness () | 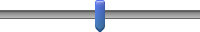 |
| --- | --- |
| Degree of Processing () | 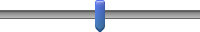 |

**This is pasta.**

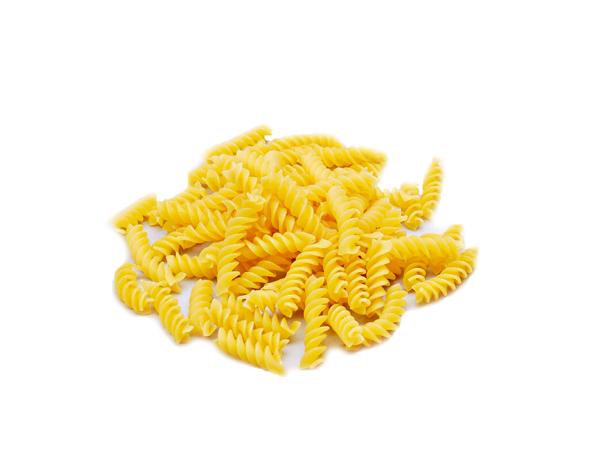


Please rank the healthfulness and degree of processing of this food on a scale from 0-10.
**Healthfulness:** 0 = not at all healthy. 10 = very healthy.
**Processing:** 0 = not at all processed. 10 = very processed.

|  | 0 | 1 | 2 | 3 | 4 | 5 | 6 | 7 | 8 | 9 | 10 |
| --- | --- | --- | --- | --- | --- | --- | --- | --- | --- | --- | --- |

| Healthfulness () | 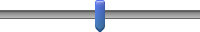 |
| --- | --- |
| Degree of Processing () | 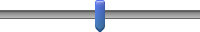 |

**This is cereal.**

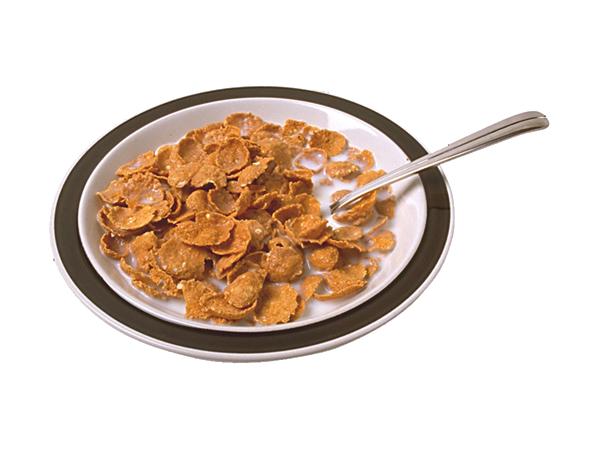


Please rank the healthfulness and degree of processing of this food on a scale from 0-10.
**Healthfulness:** 0 = not at all healthy. 10 = very healthy.
**Processing:** 0 = not at all processed. 10 = very processed.

|  | 0 | 1 | 2 | 3 | 4 | 5 | 6 | 7 | 8 | 9 | 10 |
| --- | --- | --- | --- | --- | --- | --- | --- | --- | --- | --- | --- |

| Healthfulness () | 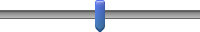 |
| --- | --- |
| Degree of Processing () | 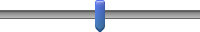 |

**This is store-bought orange juice.**

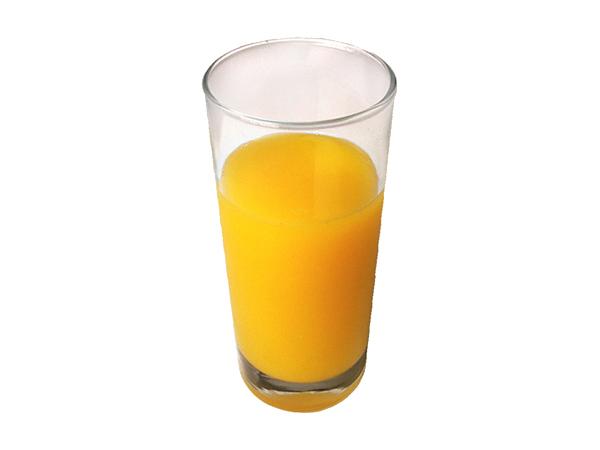


Please rank the healthfulness and degree of processing of this food on a scale from 0-10.
**Healthfulness:** 0 = not at all healthy. 10 = very healthy.
**Processing:** 0 = not at all processed. 10 = very processed.

|  | 0 | 1 | 2 | 3 | 4 | 5 | 6 | 7 | 8 | 9 | 10 |
| --- | --- | --- | --- | --- | --- | --- | --- | --- | --- | --- | --- |

| Healthfulness () | 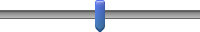 |
| --- | --- |
| Degree of Processing () | 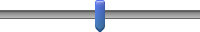 |

**This is a granola bar.**

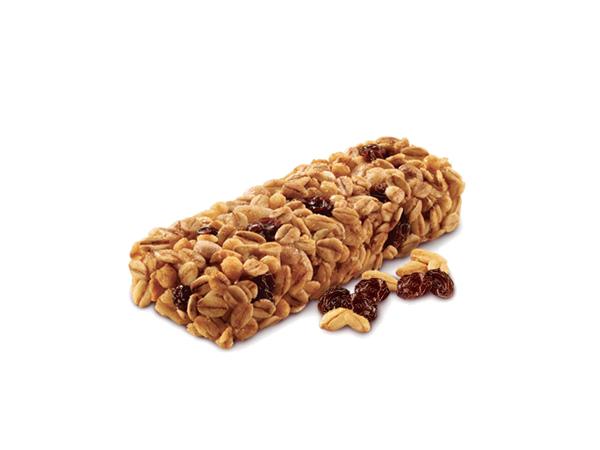


Please rank the healthfulness and degree of processing of this food on a scale from 0-10.
**Healthfulness:** 0 = not at all healthy. 10 = very healthy.
**Processing:** 0 = not at all processed. 10 = very processed.

|  | 0 | 1 | 2 | 3 | 4 | 5 | 6 | 7 | 8 | 9 | 10 |
| --- | --- | --- | --- | --- | --- | --- | --- | --- | --- | --- | --- |

| Healthfulness () | 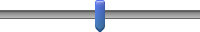 |
| --- | --- |
| Degree of Processing () | 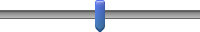 |

**This is a whole wheat bread.**

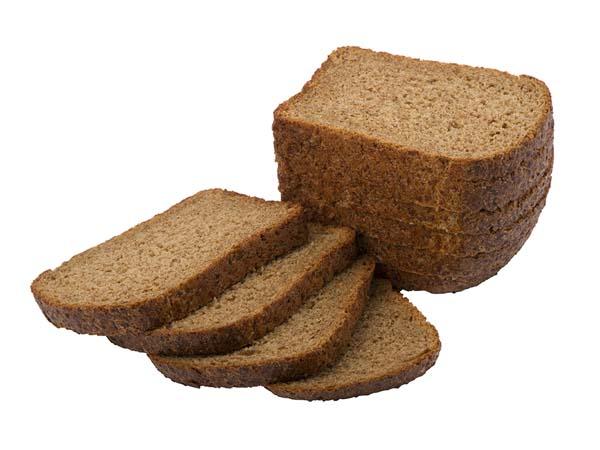


Please rank the healthfulness and degree of processing of this food on a scale from 0-10.
**Healthfulness**: 0 = not at all healthy. 10 = very healthy.
**Processing**: 0 = not at all processed. 10 = very processed.

|  | 0 | 1 | 2 | 3 | 4 | 5 | 6 | 7 | 8 | 9 | 10 |
| --- | --- | --- | --- | --- | --- | --- | --- | --- | --- | --- | --- |

| Healthfulness () | 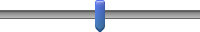 |
| --- | --- |
| Degree of processing () | 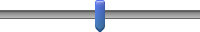 |

**This is blueberry yogurt.**


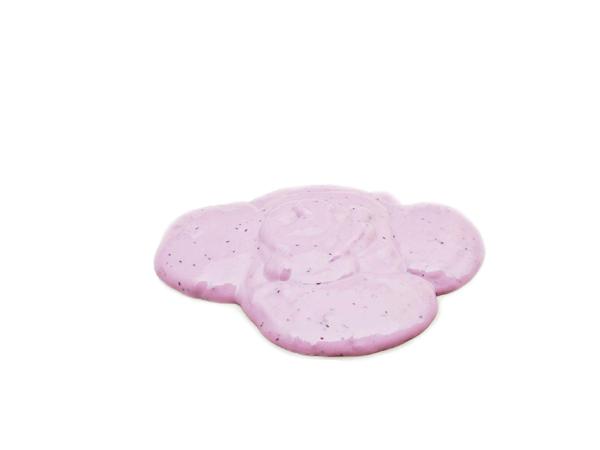


Please rank the healthfulness and degree of processing of this food on a scale from 0-10.
**Healthfulness:** 0 = not at all healthy. 10 = very healthy.
**Processing:** 0 = not at all processed. 10 = very processed.

|  | 0 | 1 | 2 | 3 | 4 | 5 | 6 | 7 | 8 | 9 | 10 |
| --- | --- | --- | --- | --- | --- | --- | --- | --- | --- | --- | --- |

| Healthfulness () | 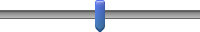 |
| --- | --- |
| Degree of Processing () | 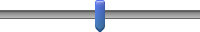 |

**These are rice cakes.**

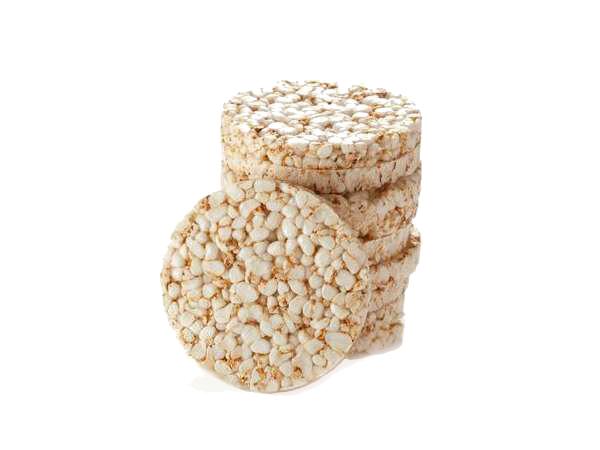


Please rank the healthfulness and degree of processing of this food on a scale from 0-10.
**Healthfulness:** 0 = not at all healthy. 10 = very healthy.

**Processing:** 0 = not at all processed. 10 = very processed.

|  | 0 | 1 | 2 | 3 | 4 | 5 | 6 | 7 | 8 | 9 | 10 |
| --- | --- | --- | --- | --- | --- | --- | --- | --- | --- | --- | --- |

| Healthfulness () | 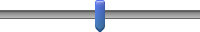 |
| --- | --- |
| Degree of Processing () | 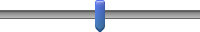 |

**This is dark chocolate.**

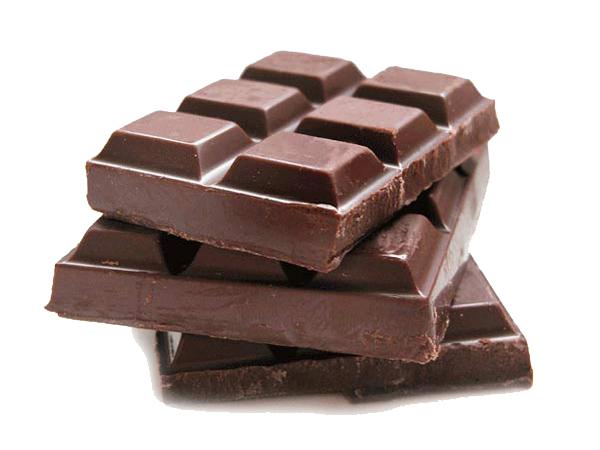


Please rank the healthfulness and degree of processing of this food on a scale from 0-10.
**Healthfulness:** 0 = not at all healthy. 10 = very healthy.
**Processing**: 0 = not at all processed. 10 = very processed.

|  | 0 | 1 | 2 | 3 | 4 | 5 | 6 | 7 | 8 | 9 | 10 |
| --- | --- | --- | --- | --- | --- | --- | --- | --- | --- | --- | --- |

| Healthfulness () | 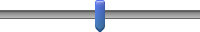 |
| --- | --- |
| Degree of Processing () | 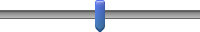 |

**This is canned fruit packed in juice.**

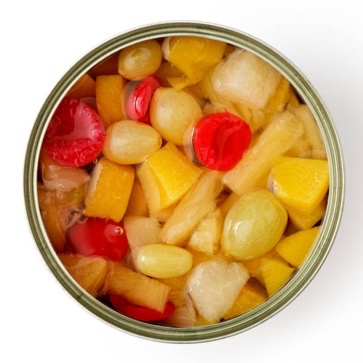


Please rank the healthfulness and degree of processing of this food on a scale from 0-10.
**Healthfulness:** 0 = not at all healthy. 10 = very healthy.
**Processing:** 0 = not at all processed. 10 = very processed.

|  | 0 | 1 | 2 | 3 | 4 | 5 | 6 | 7 | 8 | 9 | 10 |
| --- | --- | --- | --- | --- | --- | --- | --- | --- | --- | --- | --- |

| Healthfulness () | 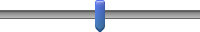 |
| --- | --- |
| Degree of Processing () | 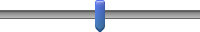 |

**These are canned tomatoes.**


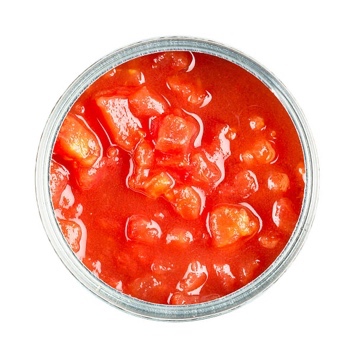


Please rank the healthfulness and degree of processing of this food on a scale from 0-10.
**Healthfulness:** 0 = not at all healthy. 10 = very healthy.

**Processing:** 0 = not at all processed. 10 = very processed.

|  | 0 | 1 | 2 | 3 | 4 | 5 | 6 | 7 | 8 | 9 | 10 |
| --- | --- | --- | --- | --- | --- | --- | --- | --- | --- | --- | --- |

| Healthfulness () | 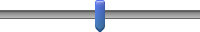 |
| --- | --- |
| Degree of Processing () | 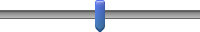 |

**This is store-bought applesauce.**

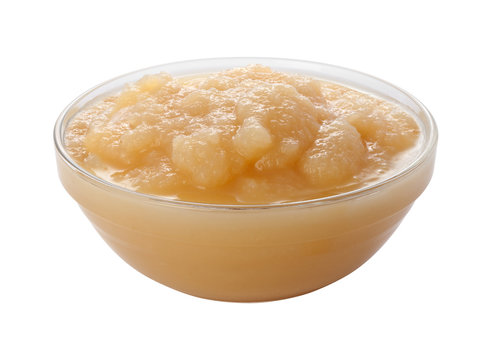


Please rank the healthfulness and degree of processing of this food on a scale from 0-10.
**Healthfulness:** 0 = not at all healthy. 10 = very healthy.
**Processing:** 0 = not at all processed. 10 = very processed.

|  | 0 | 1 | 2 | 3 | 4 | 5 | 6 | 7 | 8 | 9 | 10 |
| --- | --- | --- | --- | --- | --- | --- | --- | --- | --- | --- | --- |

| Healthfulness () | 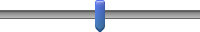 |
| --- | --- |
| Degree of Processing () | 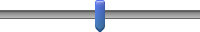 |

**This is peanut butter.**


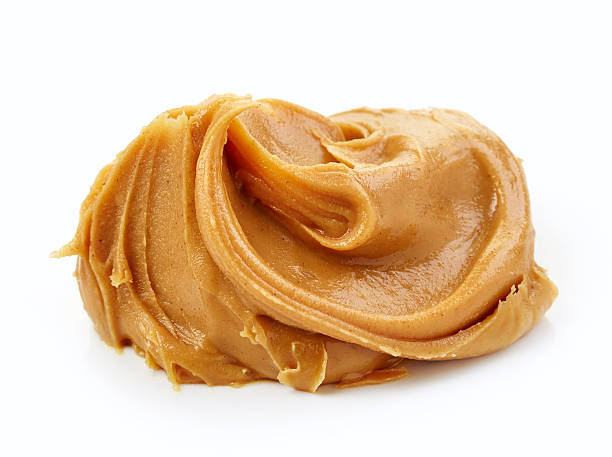


Please rank the healthfulness and degree of processing of this food on a scale from 0-10.
**Healthfulness:** 0 = not at all healthy. 10 = very healthy.
**Processing:** 0 = not at all processed. 10 = very processed.

|  | 0 | 1 | 2 | 3 | 4 | 5 | 6 | 7 | 8 | 9 | 10 |
| --- | --- | --- | --- | --- | --- | --- | --- | --- | --- | --- | --- |

| Healthfulness () | 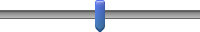 |
| --- | --- |
| Degree of Processing () | 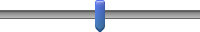 |

**This is tofu.**

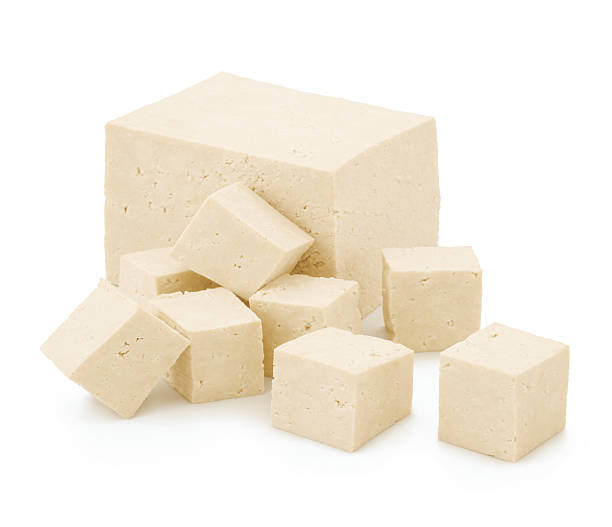


Please rank the healthfulness and degree of processing of this food on a scale from 0-10.
**Healthfulness:** 0 = not at all healthy. 10 = very healthy.
**Processing:** 0 = not at all processed. 10 = very processed.

|  | 0 | 1 | 2 | 3 | 4 | 5 | 6 | 7 | 8 | 9 | 10 |
| --- | --- | --- | --- | --- | --- | --- | --- | --- | --- | --- | --- |

| Healthfulness () | 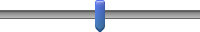 |
| --- | --- |
| Degree of Processing () | 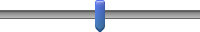 |

**This is granola.**

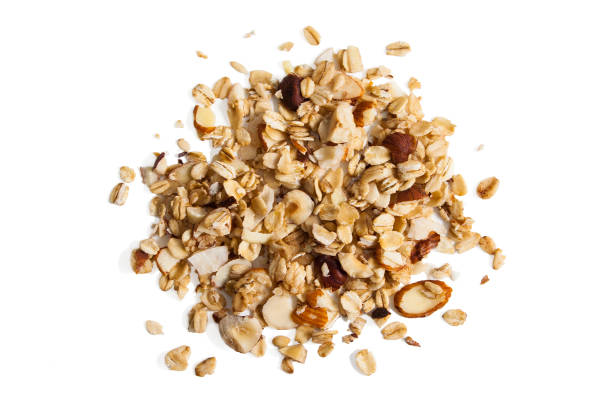


Please rank the healthfulness and degree of processing of this food on a scale from 0-10.
**Healthfulness:** 0 = not at all healthy. 10 = very healthy.

**Processing:** 0 = not at all processed. 10 = very processed.

|  | 0 | 1 | 2 | 3 | 4 | 5 | 6 | 7 | 8 | 9 | 10 |
| --- | --- | --- | --- | --- | --- | --- | --- | --- | --- | --- | --- |

| Healthfulness () | 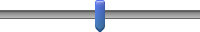 |
| --- | --- |
| Degree of Processing () | 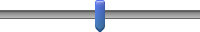 |

**This is canned chicken vegetable soup.**

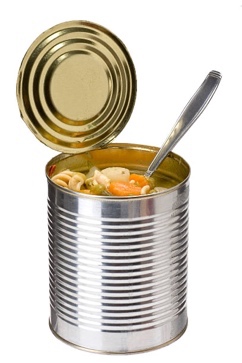


Please rank the healthfulness and degree of processing of this food on a scale from 0-10.
**Healthfulness:** 0 = not at all healthy. 10 = very healthy.
**Processing:** 0 = not at all processed. 10 = very processed.

|  | 0 | 1 | 2 | 3 | 4 | 5 | 6 | 7 | 8 | 9 | 10 |
| --- | --- | --- | --- | --- | --- | --- | --- | --- | --- | --- | --- |

| Healthfulness () | 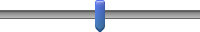 |
| --- | --- |
| Degree of Processing () | 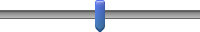 |

**This is canned tuna.**

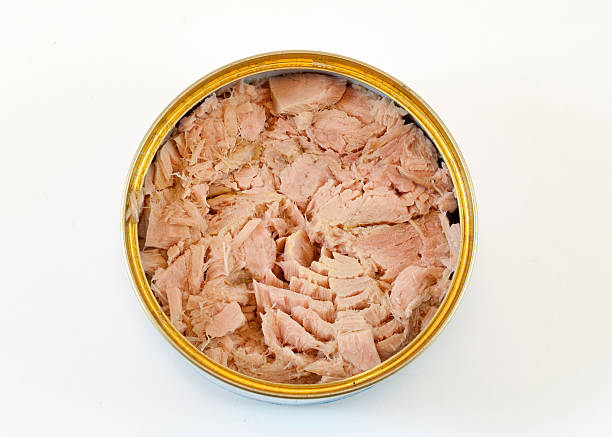


Please rank the healthfulness and degree of processing of this food on a scale from 0-10.
**Healthfulness:** 0 = not at all healthy. 10 = very healthy.
**Processing**: 0 = not at all processed. 10 = very processed.

|  | 0 | 1 | 2 | 3 | 4 | 5 | 6 | 7 | 8 | 9 | 10 |
| --- | --- | --- | --- | --- | --- | --- | --- | --- | --- | --- | --- |

| Healthfulness () | 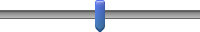 |
| --- | --- |
| Degree of Processing () | 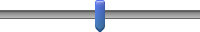 |

**This is roasted chicken.**

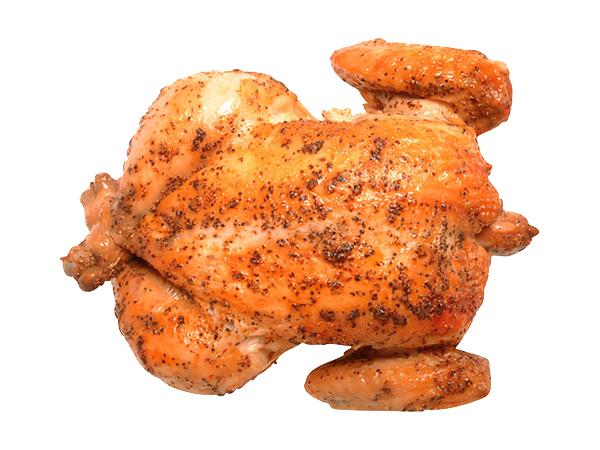


Please rank the healthfulness and degree of processing of this food on a scale from 0-10.
**Healthfulness**: 0 = not at all healthy. 10 = very healthy.

**Processing**: 0 = not at all processed. 10 = very processed.

|  | 0 | 1 | 2 | 3 | 4 | 5 | 6 | 7 | 8 | 9 | 10 |
| --- | --- | --- | --- | --- | --- | --- | --- | --- | --- | --- | --- |

| Healthfulness () | 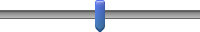 |
| --- | --- |
| Degree of Processing () | 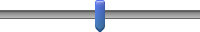 |

**This is steak.**

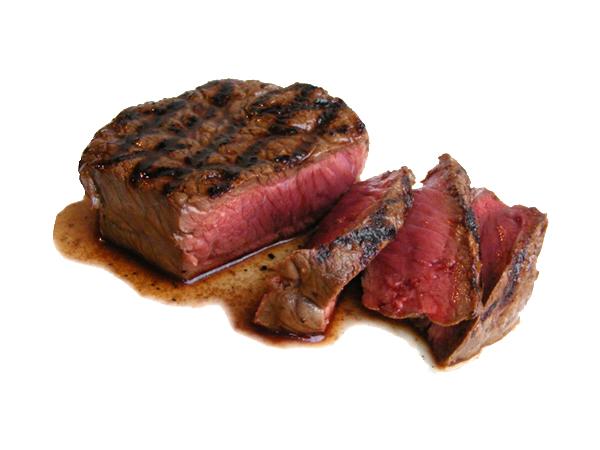


Please rank the healthfulness and degree of processing of this food on a scale from 0-10.
**Healthfulness:** 0 = not at all healthy. 10 = very healthy.
**Processing:** 0 = not at all processed. 10 = very processed.

|  | 0 | 1 | 2 | 3 | 4 | 5 | 6 | 7 | 8 | 9 | 10 |
| --- | --- | --- | --- | --- | --- | --- | --- | --- | --- | --- | --- |

| Healthfulness () | 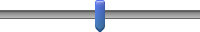 |
| --- | --- |
| Degree of Processing () | 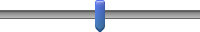 |

**These are eggs.**

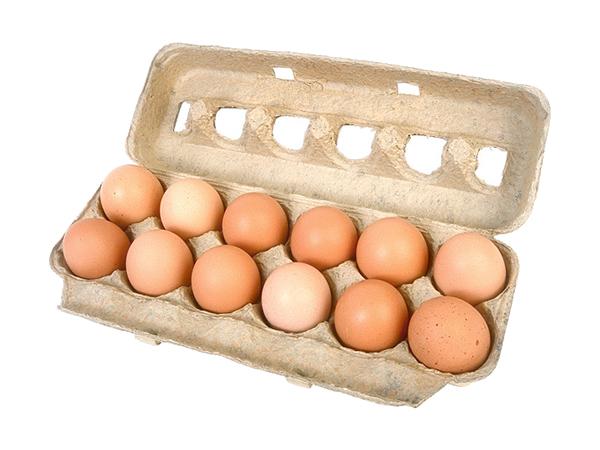


Please rank the healthfulness and degree of processing of this food on a scale from 0-10.

**Healthfulness:** 0 = not at all healthy. 10 = very healthy.

**Processing:** 0 = not at all processed. 10 = very processed.

|  | 0 | 1 | 2 | 3 | 4 | 5 | 6 | 7 | 8 | 9 | 10 |
| --- | --- | --- | --- | --- | --- | --- | --- | --- | --- | --- | --- |

| Healthfulness () | 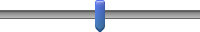 |
| --- | --- |
| Degree of Processing () | 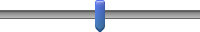 |

**These are homemade blueberry muffins.**

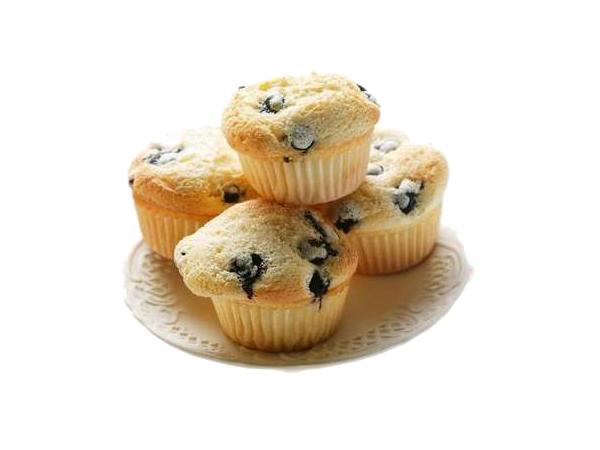


Please rank the healthfulness and degree of processing of this food on a scale from 0-10.

**Healthfulness:** 0 = not at all healthy. 10 = very healthy.

**Processing:** 0 = not at all processed. 10 = very processed.

|  | 0 | 1 | 2 | 3 | 4 | 5 | 6 | 7 | 8 | 9 | 10 |
| --- | --- | --- | --- | --- | --- | --- | --- | --- | --- | --- | --- |

| Healthfulness () | 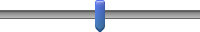 |
| --- | --- |
| Degree of Processing () | 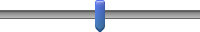 |

**These are roasted potatoes.**

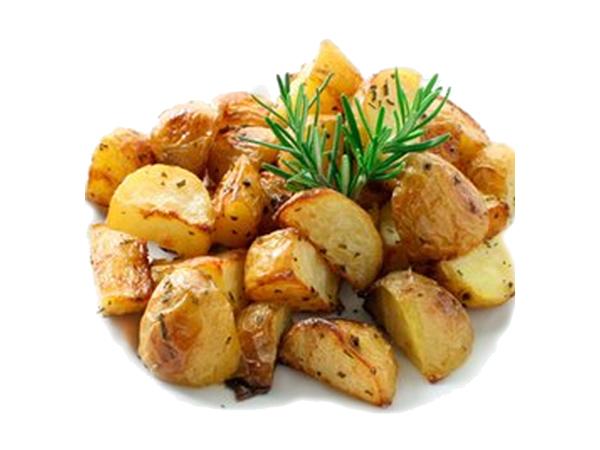


Please rank the healthfulness and degree of processing of this food on a scale from 0-10.

**Healthfulness:** 0 = not at all healthy. 10 = very healthy.

**Processing**: 0 = not at all processed. 10 = very processed.

|  | 0 | 1 | 2 | 3 | 4 | 5 | 6 | 7 | 8 | 9 | 10 |
| --- | --- | --- | --- | --- | --- | --- | --- | --- | --- | --- | --- |

| Healthfulness () | 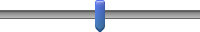 |
| --- | --- |
| Degree of Processing () | 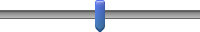 |

**This is white rice.**

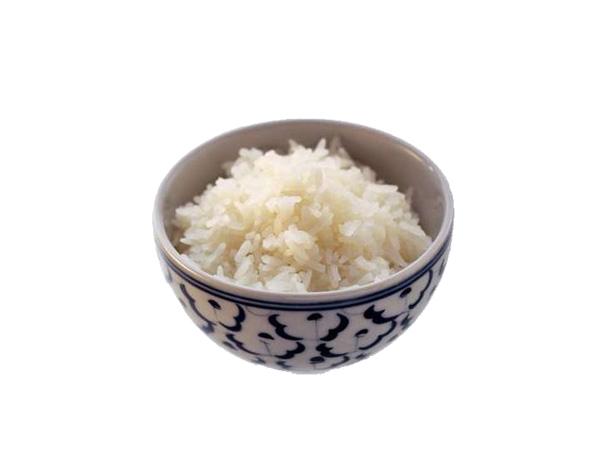


Please rank the healthfulness and degree of processing of this food on a scale from 0-10.
**Healthfulness:** 0 = not at all healthy. 10 = very healthy.

**Processing**: 0 = not at all processed. 10 = very processed.

|  | 0 | 1 | 2 | 3 | 4 | 5 | 6 | 7 | 8 | 9 | 10 |
| --- | --- | --- | --- | --- | --- | --- | --- | --- | --- | --- | --- |

| Healthfulness () | 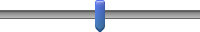 |
| --- | --- |
| Degree of Processing () | 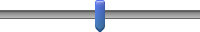 |

**This is cheese.**

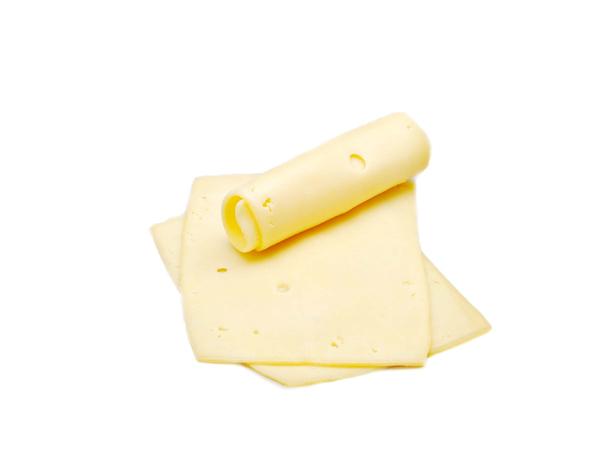


Please rank the healthfulness and degree of processing of this food on a scale from 0-10.
**Healthfulness:** 0 = not at all healthy. 10 = very healthy.
**Processing:** 0 = not at all processed. 10 = very processed.

|  | 0 | 1 | 2 | 3 | 4 | 5 | 6 | 7 | 8 | 9 | 10 |
| --- | --- | --- | --- | --- | --- | --- | --- | --- | --- | --- | --- |

| Healthfulness () | 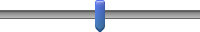 |
| --- | --- |
| Degree of Processing () | 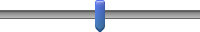 |

**This is whole milk.**

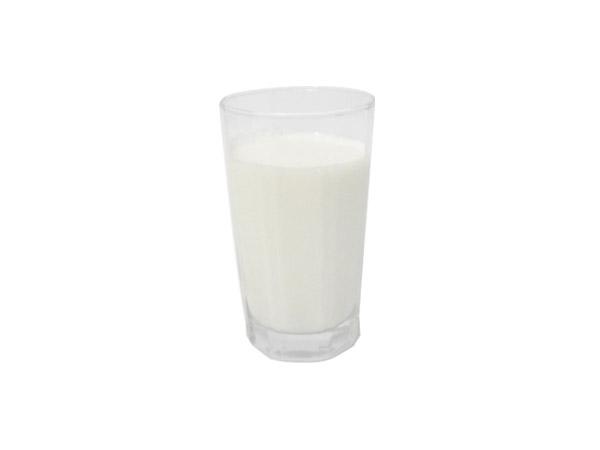


Please rank the healthfulness and degree of processing of this food on a scale from 0-10.

**Healthfulness**: 0 = not at all healthy. 10 = very healthy.
**Processing:** 0 = not at all processed. 10 = very processed.

|  | 0 | 1 | 2 | 3 | 4 | 5 | 6 | 7 | 8 | 9 | 10 |
| --- | --- | --- | --- | --- | --- | --- | --- | --- | --- | --- | --- |

| Healthfulness () | 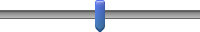 |
| --- | --- |
| Degree of Processing () | 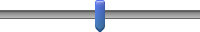 |

**These are cashews.**

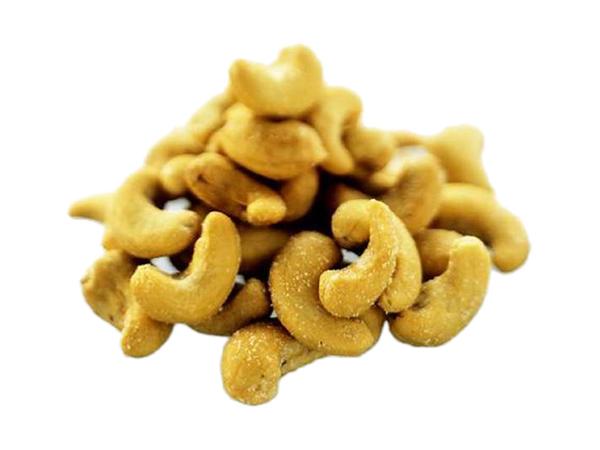


Please rank the healthfulness and degree of processing of this food on a scale from 0-10.

**Healthfulness:** 0 = not at all healthy. 10 = very healthy.

**Processing:** 0 = not at all processed. 10 = very processed.

|  | 0 | 1 | 2 | 3 | 4 | 5 | 6 | 7 | 8 | 9 | 10 |
| --- | --- | --- | --- | --- | --- | --- | --- | --- | --- | --- | --- |

| Healthfulness () | 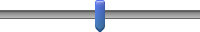 |
| --- | --- |
| Degree of Processing () | 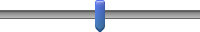 |

**These are blueberries.**

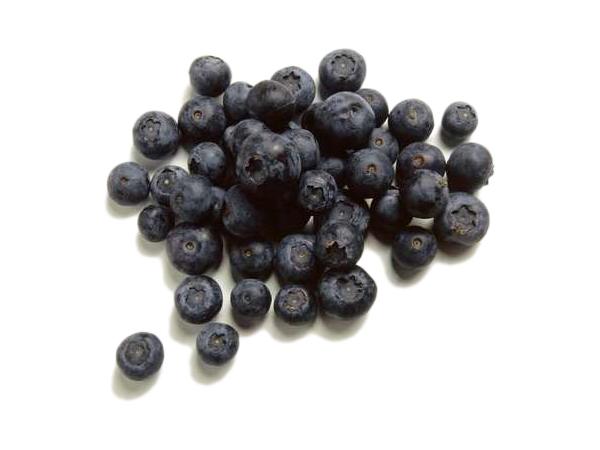


Please rank the healthfulness and degree of processing of this food on a scale from 0-10.
**Healthfulness:** 0 = not at all healthy. 10 = very healthy.

**Processing**: 0 = not at all processed. 10 = very processed.

|  | 0 | 1 | 2 | 3 | 4 | 5 | 6 | 7 | 8 | 9 | 10 |
| --- | --- | --- | --- | --- | --- | --- | --- | --- | --- | --- | --- |

| Healthfulness () | 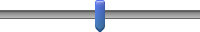 |
| --- | --- |
| Degree of Processing () | 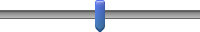 |

**These are cooked vegetables.**

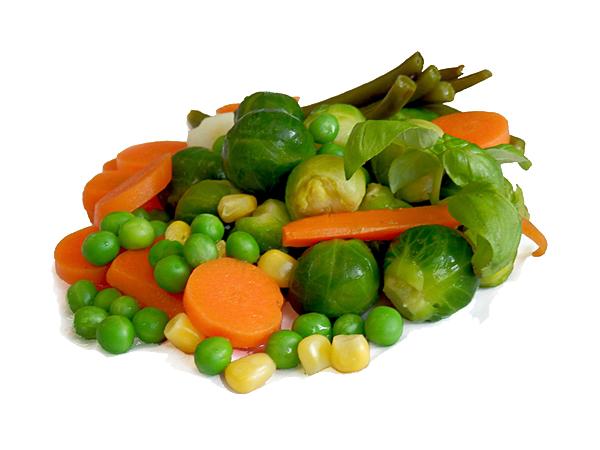


Please rank the healthfulness and degree of processing of this food on a scale from 0-10.

**Healthfulness**: 0 = not at all healthy. 10 = very healthy.

**Processing**: 0 = not at all processed. 10 = very processed.

|  | 0 | 1 | 2 | 3 | 4 | 5 | 6 | 7 | 8 | 9 | 10 |
| --- | --- | --- | --- | --- | --- | --- | --- | --- | --- | --- | --- |

| Healthfulness () | 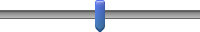 |
| --- | --- |
| Degree of Processing () | 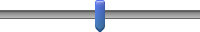 |

**This is broccoli.**

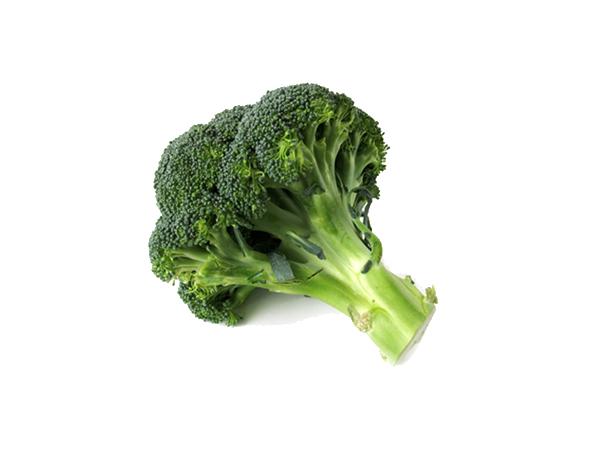


Please rank the healthfulness and degree of processing of this food on a scale from 0-10.

**Healthfulness**: 0 = not at all healthy. 10 = very healthy. **Processing:** 0 = not at all processed. 10 = very processed.

|  | 0 | 1 | 2 | 3 | 4 | 5 | 6 | 7 | 8 | 9 | 10 |
| --- | --- | --- | --- | --- | --- | --- | --- | --- | --- | --- | --- |

| Healthfulness () | 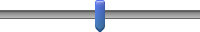 |
| --- | --- |
| Degree of Processing () | 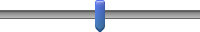 |

**This is homemade bread.**


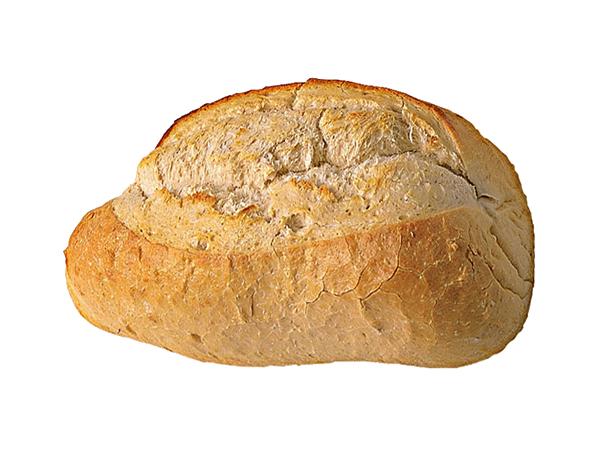


Please rank the healthfulness and degree of processing of this food on a scale from 0-10.

**Healthfulness:** 0 = not at all healthy. 10 = very healthy.

**Processing:** 0 = not at all processed. 10 = very processed.

|  | 0 | 1 | 2 | 3 | 4 | 5 | 6 | 7 | 8 | 9 | 10 |
| --- | --- | --- | --- | --- | --- | --- | --- | --- | --- | --- | --- |

| Healthfulness () | 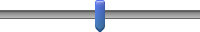 |
| --- | --- |
| Degree of Processing () | 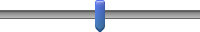 |

**This is an apple.**


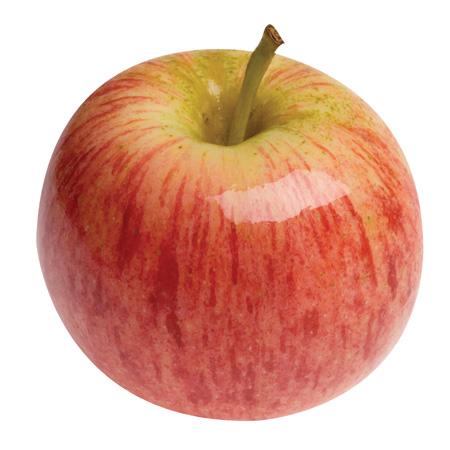


Please rank the healthfulness and degree of processing of this food on a scale from 0-10.

**Healthfulness:** 0 = not at all healthy. 10 = very healthy.

**Processing:** 0 = not at all processed. 10 = very processed.

|  | 0 | 1 | 2 | 3 | 4 | 5 | 6 | 7 | 8 | 9 | 10 |
| --- | --- | --- | --- | --- | --- | --- | --- | --- | --- | --- | --- |

| Healthfulness () |  |
| --- | --- |
| Degree of Processing () |  |

**This is plain yogurt.**

Please rank the healthfulness and degree of processing of this food on a scale from 0-10.

**Healthfulness:** 0 = not at all healthy. 10 = very healthy.

**Processing**: 0 = not at all processed. 10 = very processed.

|  | 0 | 1 | 2 | 3 | 4 | 5 | 6 | 7 | 8 | 9 | 10 |
| --- | --- | --- | --- | --- | --- | --- | --- | --- | --- | --- | --- |

| Healthfulness () |  |
| --- | --- |
| Degree of Processing () |  |

**This is plain oatmeal.**

Please rank the healthfulness and degree of processing of this food on a scale from 0-10.

**Healthfulness:** 0 = not at all healthy. 10 = very healthy.

**Processing:** 0 = not at all processed. 10 = very processed.

|  | 0 | 1 | 2 | 3 | 4 | 5 | 6 | 7 | 8 | 9 | 10 |
| --- | --- | --- | --- | --- | --- | --- | --- | --- | --- | --- | --- |

| Healthfulness () |  |
| --- | --- |
| Degree of Processing () |  |

**This is homemade pizza.**

Please rank the healthfulness and degree of processing of this food on a scale from 0-10.

**Healthfulness:** 0 = not at all healthy. 10 = very healthy.

**Processing:** 0 = not at all processed. 10 = very processed.

|  | 0 | 1 | 2 | 3 | 4 | 5 | 6 | 7 | 8 | 9 | 10 |
| --- | --- | --- | --- | --- | --- | --- | --- | --- | --- | --- | --- |

| Healthfulness () |  |
| --- | --- |
| Degree of Processing () |  |

**These are homemade sugar cookies.**

Please rank the healthfulness and degree of processing of this food on a scale from 0-10.

**Healthfulness**: 0 = not at all healthy. 10 = very healthy.

**Processing**: 0 = not at all processed. 10 = very processed.

|  | 0 | 1 | 2 | 3 | 4 | 5 | 6 | 7 | 8 | 9 | 10 |
| --- | --- | --- | --- | --- | --- | --- | --- | --- | --- | --- | --- |

| Healthfulness () |  |
| --- | --- |
| Degree of Processing () |  |

**This is homemade soup.**

Please rank the healthfulness and degree of processing of this food on a scale from 0-10.
**Healthfulness:** 0 = not at all healthy. 10 = very healthy.
**Processing:** 0 = not at all processed. 10 = very processed.

|  | 0 | 1 | 2 | 3 | 4 | 5 | 6 | 7 | 8 | 9 | 10 |
| --- | --- | --- | --- | --- | --- | --- | --- | --- | --- | --- | --- |

| Healthfulness () |  |
| --- | --- |
| Degree of Processing () |  |

**This is brown rice.**

Please rank the healthfulness and degree of processing of this food on a scale from 0-10.
**Healthfulness:** 0 = not at all healthy. 10 = very healthy.
**Processing:** 0 = not at all processed. 10 = very processed.

|  | 0 | 1 | 2 | 3 | 4 | 5 | 6 | 7 | 8 | 9 | 10 |
| --- | --- | --- | --- | --- | --- | --- | --- | --- | --- | --- | --- |

| Healthfulness () |  |
| --- | --- |
| Degree of Processing () |  |

**Table A.2. FNDDS Codes and Item Description Used for the 40 Foods Displayed to Participants in Survey**

| **Food Description** | **FNDDS Food Code** | **FNDDS Item Description and Notes** |
| --- | --- | --- |
| Broccoli | 72201100 | Broccoli, raw |
| Apple | 63101000 | Apple, raw |
| Blueberries | 63203010 | Blueberries, raw |
| Cashews | 42104000 | Cashews, NFS |
| Eggs | 31101010 | Egg, whole, raw |
| Roasted chicken | 24102050 | Chicken, NS as to part, rotisserie, NS as to skin eaten |
| Orange juice | 61210000 | Orange juice, 100%, NFS |
| Plain yogurt | 11411010 | Yogurt, NS as to type of milk, plain |
| Whole milk | 11111000 | Milk, whole |
| Plain oatmeal | 56203056 | Oatmeal, regular or quick, made with water, no added fat |
| Brown rice | 56205011 | Rice, brown, cooked, NS as to fat |
| Pasta | 56130000 | Pasta, cooked |
| White rice | 56205000 | Rice, cooked, NFS |
| Steak | 21001000 | Steak, NS as to type of meat, cooked, NS as to fat eaten |
| Cooked mixed vegetables | 75311020 | Classic mixed vegetables, NS as to form, cooked |
| Roasted potatoes | 71104030 | Potato, roasted, NFS |
| Homemade white bread | 51101050 | Bread, white, made from home recipe or purchased at a bakery |
| Homemade blueberry muffins | 52306010 | Recipe not available in FNDDS for blueberry muffin. Used FNDDS recipe for Muffin, plain (52306010) to estimate proportion of each Nova group. Scores for Food Compass based on food code, 52306010. |
| Homemade sugar cookie | 53206020 | Recipe not available in FNDDS for sugar cookie. Used FNDDS recipe for Cookie, chocolate chip (53206020), but excluded weight of chocolate chips and nuts to estimate proportion of each Nova group. Scores for Food Compass based on food code, 53206020. |
| Homemade pizza | 58107050 | Recipe not available in FNDDS for homemade cheese pizza. To estimate Nova group, combined the FNDDS recipe for  Pizza, no cheese, thin crust (58107050) because this is the only pizza food code with a recipe for the pizza crust. To add cheese to the recipe, used the food code for 14620300, Topping from cheese pizza. Scores for Food Compass based on food code, 58107050. Note that the true scores for a homemade cheese pizza may be higher or lower. |
| Homemade chicken vegetable soup | 28340660 | Chicken or turkey vegetable soup, home recipe |
| Applesauce | 63101110 | Applesauce, regular |
| Canned tuna | 26155110 | Tuna, canned, NS as to oil or water pack |
| Canned tomatoes | 74201003 | Tomatoes, cooked, from canned, NS as to method |
| Cheese | 14109010 | Cheese, Swiss |
| Peanut butter | 42202000 | Peanut butter |
| Tofu | 41420010 | Soybean curd |
| Granola | 57227000 | Cereal, granola |
| Rice cakes | 54318500 | Rice cake |
| Whole wheat bread | 51300110 | Bread, whole wheat |
| Dark chocolate | 91705300 | Chocolate, sweet or dark |
| Blueberry yogurt | 11430000 | Yogurt, NS as to type of milk, fruit |
| Granola bar | 53712100 | Cereal or Granola bar, NFS |
| Canned fruit in juice | 63311170 | Fruit cocktail, canned, juice pack |
| Cereal | 57100100 | Cereal, ready-to-eat, NFS |
| White bread | 51101000 | Bread, white |
| Deli ham | 25230210 | Ham, prepackaged or deli, luncheon meat |
| Canned chicken vegetable soup | 28340600 | Chicken or turkey vegetable soup, canned, prepared with water or ready-to-serve |
| Donut | 53520000 | Doughnut, NFS |
| Store-bought pizza | 58106205 | Pizza, cheese, from frozen, thick crust |

**Table A.3. Objective Nova Category, Food Compass Score, Mean Perceived Processing, and Mean Perceived Healthfulness of 40 Foods Displayed to Participants in Survey**^a,b^

| **Food Description** | **Nova Category** | **Food Compass Score** | **Mean Perceived Processing Score (SD)** | **Mean Perceived Healthfulness Score (SD)** |
| --- | --- | --- | --- | --- |
| Broccoli | 100% Nova 1 | 100 | 2.4 (3.2) | 9.1 (1.8) |
| Apple | 100% Nova 1 | 92 | 2.5 (3.3) | 9.1 (1.7) |
| Blueberries | 100% Nova 1 | 95 | 2.5 (3.3) | 9.1 (1.7) |
| Cashews | 100% Nova 1 | 90 | 3.9 (2.9) | 7.9 (2.0) |
| Eggs | 100% Nova 1 | 59 | 3.3 (3.2) | 8.1 (2.1) |
| Roasted chicken | 100% Nova 1 | 50 | 4.5 (2.8) | 7.5 (2.0) |
| Orange juice | 100% Nova 1 | 73 | 5.9 (2.6) | 6.9 (2.4) |
| Plain yogurt | 100% Nova 1 | 83 | 4.8 (2.7) | 8.0 (1.9) |
| Whole milk | 100% Nova 1 | 46 | 5.2 (2.9) | 7.4 (2.4) |
| Plain oatmeal | 99.9% Nova 1  0.1% Nova 2 | 77 | 4.2 (2.8) | 8.2 (2.0) |
| Brown rice | 99.5% Nova 1  0.5% Nova 2 | 65 | 4.0 (2.9) | 7.9 (2.1) |
| Pasta | 99.4% Nova 1  0.6% Nova 2 | 21 | 5.9 (2.5) | 5.7 (2.4) |
| White rice | 99.4% Nova 1  0.6% Nova 2 | 7 | 4.8 (1.9) | 6.4 (2.6) |
| Steak | 99.2% Nova 1  0.8% Nova 2 | 33 | 4.2 (2.9) | 6.4 (2.5) |
| Cooked mixed vegetables | 96.8% Nova 1  2.6% Nova 2  0.6% Nova 4 | 55 | 3.6 (3.1) | 8.6 (1.8) |
| Roasted potatoes | 94.9% Nova 1  4.2% Nova 2  0.9% Nova 4 | 53 | 3.8 (2.9) | 7.1 (2.2) |
| Homemade white bread | 94.4% Nova 1  4.6% Nova 2  0.9% Nova 4 | 4 | 4.4 (2.9) | 6.8 (2.3) |
| Homemade blueberry muffins | 63.1% Nova 1  36.8% Nova 2 | 13 | 5.3 (2.8) | 5.6 (2.5) |
| Homemade sugar cookie | 57.9% Nova 1  42.1% Nova 2 | 6 | 5.2 (2.9) | 3.6 (2.6) |
| Homemade pizza | 48% Nova 1  9.4% Nova 2  22.4% Nova 3  20.1% Nova 4 | 27 | 5.1 (2.7) | 6.1 (2.4) |
| Homemade chicken vegetable soup^c^ | 41.5% Nova 1  5.6% Nova 2  58.0% Nova 4 | 62 | 3.8 (3.0) | 8.4 (1.8) |
| Applesauce | 100% Nova 3 | 51 | 6.1 (2.5) | 6.3 (2.3) |
| Canned tuna | 100% Nova 3 | 84 | 5.7 (2.7) | 7.2 (2.3) |
| Canned tomatoes | 100% Nova 3 | 100 | 5.9 (2.5) | 6.6 (2.3) |
| Cheese | 100% Nova 3 | 37 | 6.0 (2.5) | 6.6 (2.3) |
| Peanut butter | 100% Nova 3 | 73 | 5.8 (2.5) | 6.9 (2.3) |
| Tofu | 100% Nova 3 | 97 | 5.5 (3.8) | 6.8 (2.6) |
| Granola | 100% Nova 4 | 56 | 4.7 (2.8) | 7.8 (2.1) |
| Rice cakes | 100% Nova 4 | 65 | 5.4 (2.7) | 6.5 (2.6) |
| Whole wheat bread | 100% Nova 4 | 64 | 5.4 (2.6) | 7.6 (2.1) |
| Dark chocolate | 100% Nova 4 | 21 | 5.5 (2.6) | 6.2 (2.7) |
| Blueberry yogurt | 100% Nova 4 | 53 | 5.8 (2.5) | 7.0 (2.2) |
| Granola bar | 100% Nova 4 | 39 | 6.0 (2.5) | 6.5 (2.4) |
| Canned fruit in juice | 100% Nova 4 | 63 | 6.1 (2.6) | 6.0 (2.5) |
| Cereal | 100% Nova 4 | 67 | 6.4 (2.4) | 5.8 (2.5) |
| White bread | 100% Nova 4 | 6 | 6.6 (2.6) | 4.6 (2.8) |
| Deli ham | 100% Nova 4 | 16 | 6.7 (2.6) | 5.0 (2.6) |
| Canned chicken vegetable soup | 100% Nova 4 | 49 | 6.8 (2.4) | 5.7 (2.4) |
| Donut | 100% Nova 4 | 1 | 6.9 (3.0) | 2.3 (2.7) |
| Store-bought pizza | 100% Nova 4 | 21 | 7.1 (2.6) | 3.7 (2.6) |

1. Nova category and Food Compass 2.0 score obtained based on the FNDDS food code and description provided in Table A.1.
2. For a given food, participants were shown a picture of the food and were asked to rate the healthfulness and degree of processing of each food. Processing and healthfulness were ranked on a scale from 0-10, where 0 corresponded to “not at all processed” or “not at all healthy” and 10 corresponded to “very processed” or “very healthy.” Mean scores were estimated only using scores provided by participants who viewed a given item.

A high proportion of calories from Food Code 28340660 (“Homemade chicken vegetable soup”) was classified as Nova Group 4 because one of the underlying ingredients, SR code 6194 (“Soup, chicken broth, ready-to-serve”), was classified as Nova Group 4.

**Table A.4. Definition of ultra-processed foods among national sample of US adults stratified by demographic characteristics (n=4455)**

|  | **Ultra-Processed Foods Are:** | | | | | | | | | |
| --- | --- | --- | --- | --- | --- | --- | --- | --- | --- | --- |
|  | **Foods composed with more than 5 ingredients** | | **Food products submitted to a series of industrial processing** | | **Genetically modified products** | | **Food products that contain artificial ingredients** | | **I don't know what ultra-processed foods are** | |
|  | **N (%)** | **p-value** | **N (%)** | **p-value** | **N (%)** | **p-value** | **N (%)** | **p-value** | **N (%)** | **p-value** |
| **Overall** | 1175 |  | 2423 |  | 1459 |  | 2315 |  | 1053 |  |
| **Gender** |  |  |  |  |  |  |  |  |  |  |
| Male | 509 (43.3%) | 0.20 | 1112 (45.9%) | 0.19 | 609 (41.7%) | 0.001 | 1000 (43.2%) | 0.001 | 469 (44.5%) | 0.29 |
| Female | 657 (55.9%) |  | 1287 (53.1%) |  | 835 (57.2%) |  | 1291 (55.8%) |  | 579 (55.0%) |  |
| Non-binary or Other | 9 (0.8%) |  | 24 (1.0%) |  | 15 (1.0%) |  | 24 (1.0%) |  | 5 (0.5%) |  |
| **Age** |  |  |  |  |  |  |  |  |  |  |
| 18-29 | 177 (15.1%) | <0.001 | 347 (14.3%) | 0.027 | 290 (19.9%) | <0.001 | 368 (15.9%) | <0.001 | 131 (12.4%) | <0.001 |
| 30-39 | 209 (17.8%) |  | 349 (14.4%) |  | 267 (18.3%) |  | 360 (15.6%) |  | 120 (11.4%) |  |
| 40-49 | 216 (18.4%) |  | 378 (15.6%) |  | 267 (18.3%) |  | 397 (17.1%) |  | 165 (15.7%) |  |
| 50-59 | 239 (20.3%) |  | 499 (20.6%) |  | 255 (17.5%) |  | 467 (20.2%) |  | 243 (23.1%) |  |
| 60-69 | 162 (13.8%) |  | 370 (15.3%) |  | 166 (11.4%) |  | 330 (14.3%) |  | 183 (17.4%) |  |
| 70 and older | 172 (14.6%) |  | 480 (19.8%) |  | 214 (14.7%) |  | 393 (17.0%) |  | 211 (20.0%) |  |
| **Race and Ethnicity** |  |  |  |  |  |  |  |  |  |  |
| White | 738 (62.8%) | 0.030 | 1620 (66.9%) | <0.001 | 898 (61.5%) | 0.022 | 1479 (63.9%) | 0.027 | 677 (64.3%) | 0.076 |
| Asian | 43 (3.7%) |  | 98 (4.0%) |  | 57 (3.9%) |  | 94 (4.1%) |  | 31 (2.9%) |  |
| Black or African American | 165 (14.0%) |  | 213 (8.8%) |  | 172 (11.8%) |  | 246 (10.6%) |  | 142 (13.5%) |  |
| Native American | 13 (1.1%) |  | 24 (1.0%) |  | 18 (1.2%) |  | 27 (1.2%) |  | 15 (1.4%) |  |
| Hispanic | 169 (14.4%) |  | 357 (14.7%) |  | 243 (16.7%) |  | 363 (15.7%) |  | 155 (14.7%) |  |
| Other | 47 (4.0%) |  | 111 (4.6%) |  | 71 (4.9%) |  | 106 (4.6%) |  | 33 (3.1%) |  |
| **Income** |  |  |  |  |  |  |  |  |  |  |
| <$25,000 | 231 (19.7%) | 0.027 | 440 (18.2%) | <0.001 | 293 (20.1%) | 0.14 | 448 (19.4%) | 0.003 | 297 (28.2%) | <0.001 |
| $25,000 - < $75,000 | 526 (44.8%) |  | 1063 (43.9%) |  | 675 (46.3%) |  | 1029 (44.4%) |  | 471 (44.7%) |  |
| > $75,000 | 388 (33.0%) |  | 835 (34.5%) |  | 448 (30.7%) |  | 758 (32.7%) |  | 243 (23.1%) |  |
| Prefer not to say | 30 (2.6%) |  | 85 (3.5%) |  | 43 (2.9%) |  | 80 (3.5%) |  | 42 (4.0%) |  |
| **Education** |  |  |  |  |  |  |  |  |  |  |
| High school or less | 280 (23.8%) | 0.17 | 488 (20.1%) | <0.001 | 366 (25.1%) | 0.24 | 499 (21.6%) | <0.001 | 389 (36.9%) | <0.001 |
| Some college | 376 (32.0%) |  | 779 (32.2%) |  | 472 (32.4%) |  | 728 (31.4%) |  | 301 (28.6%) |  |
| College degree or higher | 519 (44.2%) |  | 1156 (47.7%) |  | 621 (42.6%) |  | 1088 (47.0%) |  | 363 (34.5%) |  |
| **Political Affiliation** |  |  |  |  |  |  |  |  |  |  |
| Republican | 357 (30.4%) | 0.29 | 720 (29.7%) | 0.39 | 461 (31.6%) | 0.20 | 697 (30.1%) | 0.37 | 315 (29.9%) | 0.28 |
| Democrat | 437 (37.2%) |  | 863 (35.6%) |  | 491 (33.7%) |  | 809 (34.9%) |  | 359 (34.1%) |  |
| Independent | 381 (32.4%) |  | 840 (34.7%) |  | 507 (34.7%) |  | 809 (34.9%) |  | 379 (36.0%) |  |
| **Household size** |  |  |  |  |  |  |  |  |  |  |
| 1 person | 271 (23.1%) | 0.009 | 577 (23.8%) | 0.092 | 303 (20.8%) | <0.001 | 516 (22.3%) | 0.13 | 255 (24.2%) | 0.42 |
| 2-3 people | 600 (51.1%) |  | 1325 (54.7%) |  | 760 (52.1%) |  | 1249 (54.0%) |  | 573 (54.4%) |  |
| 4 or more | 304 (25.9%) |  | 521 (21.5%) |  | 396 (27.1%) |  | 550 (23.8%) |  | 225 (21.4%) |  |
